# Fine tuning wheat heading time through genome editing of transcription factor binding sites in *Ppd-1* gene promoter

**DOI:** 10.1038/s41598-025-25295-8

**Published:** 2025-11-26

**Authors:** Antonina A. Kiseleva, Ekaterina M. Timonova, Alina A. Berezhnaya, Anastasiya E. Kolozhvari, Alex V. Kochetov, Elena A. Salina

**Affiliations:** 1 The Federal State Budgetary Institution of Science Federal Research Center Institute of Cytology and Genetics, Siberian Branch of the Russian Academy of Sciences, Novosibirsk, Russia; 2 Kurchatov Genomics Center, Institute of Cytology and Genetics SB RAS, Novosibirsk, Russia; 3 Novosibirsk State Unversity, Novosibirsk, Russia; 4 Novosibirsk State Agrarian University, Novosibirsk, Russia

**Keywords:** Common wheat, Heading time, Genome editing, Photoperiod sensitivity, Ppd-1, Agricultural genetics, Plant sciences

## Abstract

Increasing the productivity and adaptability of agricultural plants often depends on optimizing heading time. To develop common wheat lines with accelerated heading and investigate its regulation, we targeted the *PPD-1* genes, which control photoperiod sensitivity. Large deletions in the promoter regions of these genes are known to disrupt their expression, resulting in early heading. Using CRISPR/Cas9 genome editing, we generated wheat plants with mutations in the promoter regions of the *Ppd-D1* and *Ppd-B1* genes. These mutations included nucleotide substitutions, deletions, and insertions ranging from several to hundreds of base pairs, occurring within probable transcription factor binding sites, that may influence gene expression. Under short-day conditions, we assessed *PPD-1* gene expression in T0 plants and T2 lines with different mutations. Our analysis revealed that deletions spanning the CHE transcription repressor binding sites altered gene expression patterns, supporting the hypothesis regarding the role of these cis-elements in regulating *PPD-1* expression. Furthermore, plants with different mutations displayed distinct diurnal expression patterns, suggesting the involvement of additional transcription factors in the regulation of this gene. Evaluation of heading time in T1 and T2 families with different mutations demonstrated that plants with mutations affecting the “core region”, including the CHE binding sites, initiated heading significantly earlier than those without mutations.

**Supplementary Information:**

The online version contains supplementary material available at 10.1038/s41598-025-25295-8.

## Introduction

The development of high-yield and environmentally adaptive wheat varieties is crucial for achieving sustainable agriculture^1^. One important tool for improving cropping intensity is shortening growth duration, as early-flowering varieties have higher per-day productivity than medium-flowering ones^2^. Thus, controlling heading time is a key factor in increasing productivity^3^.

In bread wheat, the *Photoperiod-1* (*PPD-1*) genes play a major role in regulating the duration from seedling to heading^4^. Due to the pleiotropic effects of these genes, *PPD-1* not only influence heading time but also affect traits related to plant adaptability to environmental conditions and spike architecture, which directly impact grain yield^5–7^.

*Ppd-1* regulates the transition to flowering by modifying the expression of *FLOWERING LOCUS T* (*TaFT-1*, *Vrn-3*) ^6,8,9^. The FT1 protein is transported from the leaves to the shoot apical meristem, where it binds to FLOWERING LOCUS D-LIKE (TaFDL2) and 14-3-3 proteins, forming a “florigen activation complex” (FAC)^10^. This complex regulates the expression of meristem identity genes, which promote the transition to the reproductive stage^11,12^.

The expression of *PPD-1* is supposed to be regulated by the circadian clock protein EARLY FLOWERING 3 (ELF3) via LUX ARRHYTHMO (LUX), which, in turn, is controlled by the phytochromes PhyC and PhyB that perceive light signals^13–15^. It has been demonstrated that a PHYB-PHYC heterodimer is required to repress the activity of *ELF3*^13^, and ELF3 was shown to bind directly to the *PPD1* promoter *in vivo*^15^. Wild-type photoperiod-sensitive (PS) *Ppd-1* alleles are expressed during the day (light period), with expression peaking 3 to 6 h after dawn, and remain inactive during the night (dark period). Several photoperiod insensitive (PI) alleles, designated as *Ppd-1a*, promote the transition to the heading stage^4,16,17^. The *Ppd-D1a* and *Ppd-A1a* alleles feature large deletions in their promoter regions, while *Ppd-B1a* alleles are characterized by insertion in the promoter region or an increased copy number^18^. Different PI alleles may exhibit altered expression patterns, commonly characterized by expression during the dark period^5–7^.

Previous analyses of promoter sequences reveal crucial cis-elements within the deletion regions of PI alleles, including the G-box, TATA-box, and binding sites for the transcription factor CHE (CCA1 HIKING EXPEDITION)^17,19^. CHE encodes a transcription factor of the TCP family and is hypothesized to act as a transcription repressor. Its binding site in wheat has the sequence GG[G/C]CCCAC^20^, with two such binding sites located 150 base pairs apart in the *Ppd-1* promoter^19^. It has been hypothesized that deletion (*Ppd-A1* and *Ppd-D1*) or separation (*Ppd-B1*) of CHE binding sites results in *PPD-1* misexpression and subsequent accelerated heading time^17^.

Genome editing is a pivotal modern approach for studying gene functions, their regulation, and enhancing desirable traits in crop cultivars^21^. Nevertheless, the challenge of editing the genome of non-model crop varieties, such as bread wheat, persists due to the low transformation and regeneration capacities of most cultivars^22^. Another challenge limiting the broad application of genome editing is the complex process involved in removing transgenic vector inserts from the edited mutant plants^23^.

For this genome-editing project, we selected the common wheat line Velut, known for its high productivity, resistance to several pathogens, and good regenerative capacity. However, Velut is a late-flowering line and does not consistently reach maturity under the environmental conditions of the Urals and Western Siberia, the primary regions for cultivating spring common wheat in Russia. Our objectives were to accelerate Velut’s heading time and study *PPD-1* gene regulation by obtaining plants with deletions of various lengths in the target gene promoters. Our specific objectives were to: (i) generate a range of promoter-edited alleles of *Ppd-D1* and *Ppd-B1* affecting putative transcriptional repressor binding sites; (ii) evaluate their effects on *Ppd-1* expression and heading time; and (iii) assess the feasibility of selecting transgene-free mutant lines directly from the T0 population using a standard biolistic transformation protocol. These efforts aim to expand the genetic toolkit for flowering time regulation in elite wheat cultivars and explore practical strategies for generating non-transgenic edited plants in polyploid crops.

## Results

### Target selection and SgRNA design

**Fig. 1:**
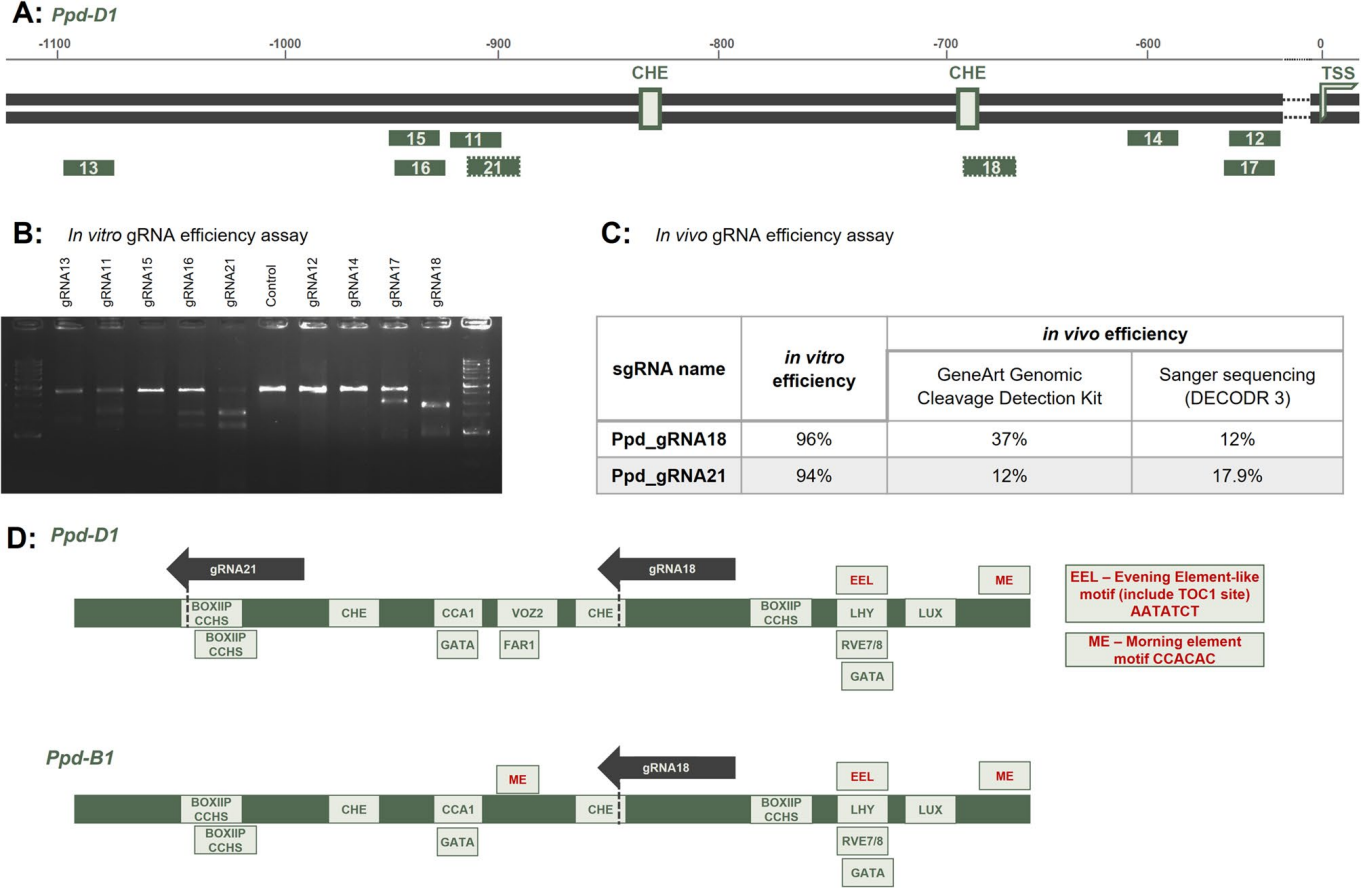
A Position of gRNAs on the target sequence of *Ppd-D1*. Light-green rectangles demonstrate hypothetic positions of CHE (CCA1 HIKING EXPEDITION) binding sites. gRNA18 and gRNA21, selected for the plasmid construction, are distinguished with dotted frame, *TSS* transcription start site; **B** electrophoresis of the *Ppd-D1* target region *in vitro* digestion by RNP complexes of Cas9 and different gRNAs to assess the efficiency of different gRNAs; **C** table, representing results of the *in vivo* gRNA efficiency estimation in protoplasts with GeneArt Genomic Cleavage Detection kit and analysis of Sanger sequences with DECODR 3.0 software; **D** position of transcription factor binding sites (TFBS), predicted in key promoter region of *Ppd-D1* and *Ppd-B1* genes. Dark gray arrows above the sequences indicate positions of gRNA18 and gRNA21, used in this study.

A comparison of known *Ppd-1a PI* alleles identified a promoter region common to all deletions, approximately 900 base pairs in length^8^. Given that the most likely regulator of *Ppd-1* expression are the CCA1 HIKING EXPEDITION (CHE) binding sites, we designed guide RNAs (gRNAs) to excise a small promoter fragment encompassing these sites. Specifically, we designed ten gRNAs to target the CHE binding sites (Fig. 1A), positioning five gRNAs on each side of the hypothetical deletion region to create small deletions overlapping this site. The sgRNAs designed for the target region with high predicted efficiency corresponded only to *Ppd-D1* and *Ppd-B1*, but not to *Ppd-A1*; therefore, we did not include *Ppd-A1* in the editing.

Sequencing the target regions of the *Ppd-D1* and *Ppd-B1* genes in the Velut line revealed no differences from the reference sequences of the *Ppd-D1* (DQ885766) and *Ppd-B1* (DQ885757) alleles. Allele-specific PCR targeting the known *Ppd-A1a.1* allele demonstrated that Velut carries a photoperiod-sensitive *Ppd-A1* allele.

### gRNA efficiency Estimation

To select sgRNAs that guide the Cas9 protein to cleave target sequences more effectively, we assessed the activity of RNP complexes with ten designed sgRNAs using an in vitro cleavage assay. Ribonucleoprotein (RNP) complexes with gRNAs 12, 14, 15, and 20 demonstrated very low cleavage ability (0–6%), while gRNAs 11, 13, 16, and 17 exhibited moderate activity (22–39%). In contrast, RNP complexes with gRNA18 and gRNA21 were highly efficient, showing nearly complete digestion of the target sequence (96% and 94%, respectively) (Fig. 1B).

Subsequently, we evaluated the activity of the selected gRNAs, gRNA18 and gRNA21, in protoplasts under *in vivo* conditions (Fig. 1C). The cleavage assay indicated that RNP complexes with these sequences demonstrated reduced efficiency in digesting the target sequence (37% and 12%, respectively) as compared to the in vitro results (Fig. 1C).

The selected gRNAs were then combined with the classic sgRNA backbone and the wheat U3 promoter and subsequently integrated with the SpCas9 and BAR cassettes from the MoClo Kit^24^.

### Editing of the *Ppd-D1* and *Ppd-B1* promoters

In our experiment to perform biolistic-mediated transformation, we used embryogenic callus induced from 931 isolated immature embryos. A single plasmid vector L2_41780_Ppd_gRNA18 + 21_Cas9_bar was used to transform the embryogenic callus of the common wheat line Velut with the PDS-1000/He Gun, aiming to generate plants with novel mutations in the promoters of the *Ppd-D1* and *Ppd-B1* genes.

Following selection on media containing phosphinothricin, we obtained 133 independent T0 plantlets. A detailed summary of the screening results for regenerated plants, both by individual experiments and as an overall average, is provided in Table 1. PCR screening with primers specific to the BAR and gRNA sequences revealed that 35 plantlets (26.3% of total amount of regenerated plants) carried the plasmid insertion (Table S3). Estimation of the number of plasmid copy insertions in the genome revealed a range of 1 to 121 copies per genome (Table S3). Next, using NGS (Next Generation Sequencing) and Sanger sequencing, we analyzed the target regions of the *Ppd-D1* and *Ppd-B1* genes. The analysis showed that 46 (35%) of the 133 plantlets contained various mutations. Of these, 26 plantlets had both plasmid insertions and mutations in the target regions, while 9 plantlets carried the plasmid but did not exhibit modifications in the target regions. An additional 20 plantlets had various mutations in the target region of the *Ppd-1* genes but lacked plasmid integration, suggesting that these mutations likely resulted from transient expression. In total, we obtained 46 primary mutant plants.

**Table 1:** Efficiency of genetic transformation and targeted mutagenesis in T0 Transgenic events of wheat line Velut.

Experiment №	Number of transformed callus	Number of regenerated plants	T0 Plants with plasmid insert (stable transformation efficiency, %)^a^	T0 Plants with mutations (mutagenesis efficiency, %)^a^	T0 Plants with mutations, plasmid insert	T0 Plants with mutations, plasmid-free
1	240	11	10 (4.16%)	9 (3.75%)	7	2
2	240	61	13 (5.42%)	23 (9.58%)	11	12
3	180	30	6 (3.33%)	9 (5%)	5	4
4	180	26	2 (1.1%)	4 (2.2%)	2	2
5	91	5	4 (4.4%)	1 (1.1%)	1	0
Total	**931**	**133**	**35 (3.76%)**	**46 (4.94%)**	**26**	**20**

Bold values indicate total data across all experiments.

^a^Efficiency of transformation and mutagenesis is presented as a percentage based on the number of transformed explants.

Sequence analysis revealed that the most common mutation was a 1 bp indel, although longer indels, ranging from 4 to 17 bp were also observed in many samples (Table S3, Supplementary file 1). Additionally, plants with large deletions that removed both potential repressor binding sites were identified. Specifically, plants V8-T0, V12-T0, V20-T0, V46-T0, V59-T0, V71-T0, and V121-T0 carried deletions in *Ppd-D1* ranging from 219 to 266 bp in length.

In plant V103-T0, a 345 bp insertion was detected at the Cas9 target site. Sequence analysis revealed that this fragment originated from the transformation vector L2_41780_Ppd_gRNA18 + 21_Cas9_bar and included sequences corresponding to the left border (LB) region of the T-DNA. This suggests that the insertion likely occurred via non-homologous end joining (NHEJ), a common DNA repair mechanism in plants that can lead to unintended integration of vector-derived sequences during genome editing.

Similarly, in plant V22-T0, two large insertions were identified – 509 bp and approximately 600 bp in length. The shorter fragment was derived from the *Ppd-D1* exon, while the longer insertion showed partial homology to the wheat U3 promoter sequence, suggesting that it also originated from the transformation vector. Both insertions are consistent with NHEJ-mediated repair events.

Most plants carried mutations in both *Ppd-D1* and *Ppd-B1* simultaneously, indicating high activity of the genome editing system used. Mutations in *Ppd-D1* were typically identified at both target sites for sgRNA-18 and sgRNA-21, suggesting strong activity of both designed sgRNAs. However, eight plants (V3-T0, V11-T0, V14-T0, V15-T0, V20-T0, V22-T0, V37-T0, V59-T0) had mutations only in *Ppd-D1*, while four plants (V10-T0, V19-T0, V27-T0, V133-T0) had mutations only in *Ppd-B1*. Only five plants (V8-T0, V66-T0, V73-T0, V112-T0, and V124-T0) were homozygous mutants for both *Ppd-D1* and *Ppd-B1*. Additionally, eleven plants (V14-T0, V18-T0, V22-T0, V25-T0, V32-T0, V33-T0, V46-T0, V52-T0, V75-T0, V146-T0, V163-T0) were putative chimeras, as they harbored more than two sequence variants.

Thus, by employing CRISPR/Cas9 genome editing techniques, we successfully engineered a series of wheat plants with diverse mutations within the promoter regions of the *PPD-1* genes. These mutations ranged from small nucleotide indels to deletions of up to 266 base pairs, targeting regions that include binding sites for various transcriptional regulators. The numbering of mutant Velut lines is consistent across generations T0, T1, and T2, indicating their common origin. For instance, seeds from the main spike of mutant V103-T0 were harvested, and 60 seeds were sown to produce the V103-T1 plants. Based on molecular genetic analysis, several V103-T1 plants were selected for seed collection, and these seeds were subsequently used to establish the V103-T2 generation.

### Expression patterns of *Ppd-1* genes with different mutations in the promoter region

In this study, we evaluated the expression of *Ppd-D1* and *Ppd-B1* in T0 plants carrying heterozygous or homozygous mutations and T2 plants carrying only homozygous mutations in the target genes. Our aim was not only to assess expression patterns in homozygous mutant lines, but also to determine whether expression changes were detectable in T0 plants and to what extent. Previous studies have shown *Ppd-1* alleles associated with photoperiod insensitivity are dominant^25^, and that heterozygosity is sufficient for their effects to manifest. It has previously been shown that expression of *Ppd-1* homoeologs is largely independent, and changes in one homoeolog (e.g., *Ppd-D1*) do not significantly influence the expression of others^16^. Therefore, the expression differences observed here are likely the result of cis-regulatory effects from the targeted promoter mutations. We selected eight T0 plants and six T2 lines with various mutations in the target genes, cultivated in a climate chamber under short-day conditions (12 h of light/12 h of darkness). For material collection, we focused on the flag leaf developmental stage and used the flag leaf as the tissue of interest.

**Fig. 2:**
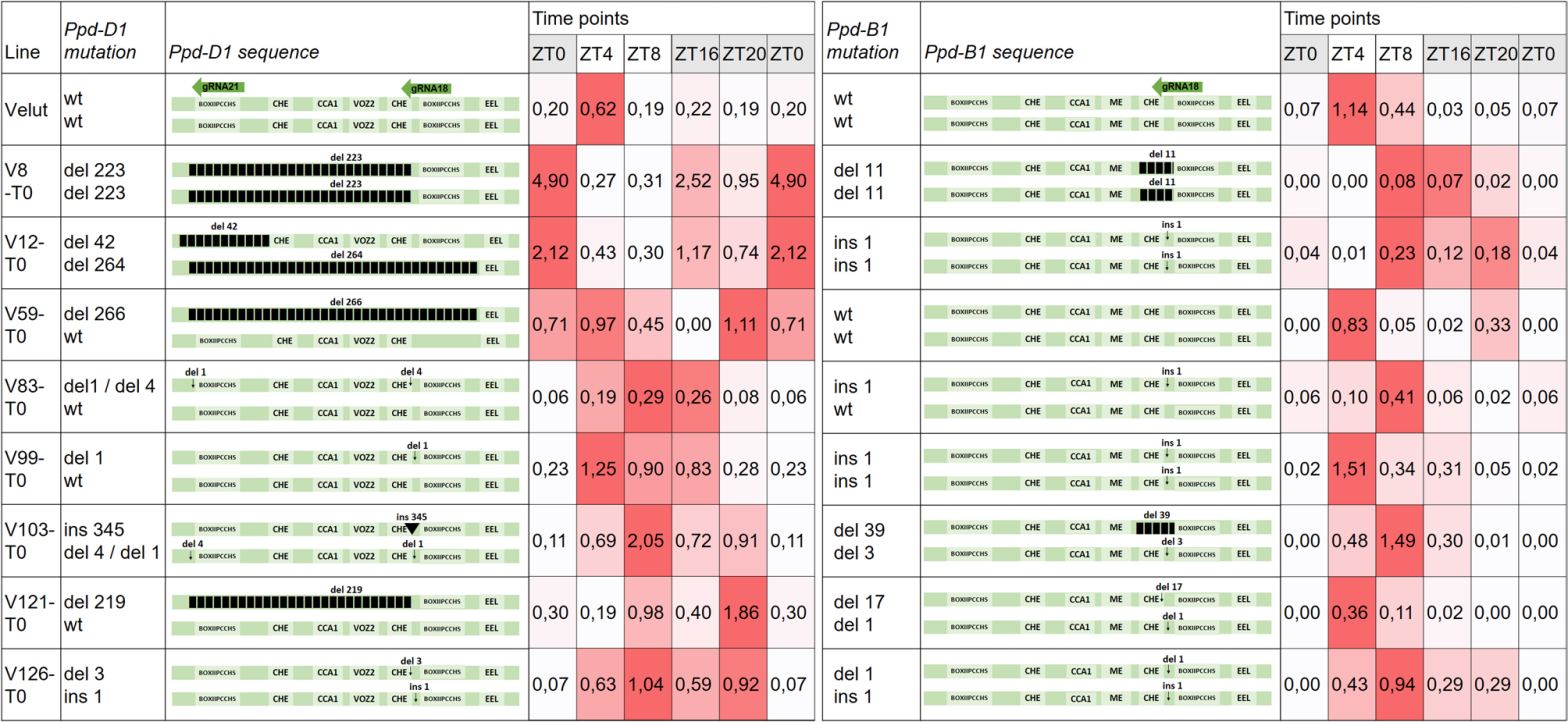
Description of mutations and the diurnal expression patterns of *Ppd-D1* and *Ppd-B1* genes in T0 plants. Gene expression levels are shown schematically using a color scale—the darker the color, the higher the level of expression at a given time point. The numbers correspond to the relative level of expression. TFBSs for CHE (CCA1 HIKING EXPEDITION), CCA1 (Circadian Clock Associated 1), ME (morning element), EEL (evening element) are indicated.

The studied T0 plants exhibited different expression patterns (Fig. 2). Velut, a wild-type (wt) control, demonstrated expression of both *Ppd-D1* and *Ppd-B1*, with peaks occurring at the 4-h light time point, consistent with previous reports that *Ppd-1* expression peaks 3–4 h after light exposure begins^9,16^. All mutations in *Ppd-D1* altered the expression pattern, with the exception of V99-T0, which harbored a 1 bp deletion downstream of the CHE binding site and exhibited an expression pattern similar to the wild type, peaking at the 4-h light time point and showing decreased expression during the dark period.

The highest level of *Ppd-D1* expression among all samples was observed in V8-T0, which peaked at dawn, just before the light was turned on. V8-T0 contained a long deletion of 223 bp in the target region of the promoter. Similarly, V12-T0, which exhibited a 264 bp deletion, displayed a comparable diurnal expression pattern, with its highest expression at the ZT0 time point. Unlike the wild-type allele, V12-T0 showed high expression during the night (ZT16) and almost no expression during the light period.

In contrast, V59-T0 and V121-T0, which also harbored large deletions of 266 bp and 219 bp, respectively, showed expression patterns distinct from both the wild-type and those observed in V8-T0 and V12-T0. V59-T0 exhibited relatively uniform expression at ZT0, ZT4, and ZT20, without a well-defined peak, and showed no expression at ZT16.

V83-T0, V103-T0, and V126-T0 exhibited similar expression patterns for *Ppd-D1*, with peaks shifted to the latter part of the day and during the night. V83-T0 and V126-T0 contained small indels affecting one of the CHE binding sites. In contrast, V103-T0 possessed two types of mutations: small indels similar to those in V83-T0 and V126-T0, as well as a large insertion of 345 bp located downstream of both CHE binding sites and the EEL site (Fig. 2).

For *Ppd-B1* expression patterns, V59-T0, V99-T0, and V121-T0 were similar to the wild type. V59-T0 contained the wild-type *Ppd-B1* sequence, V99-T0 had a 1 bp insertion that did not affect the CHE binding site, and V121-T0 exhibited a 17 bp deletion that removed two bp from the CHE binding site.

In contrast, other samples—V12-T0, V83-T0, V103-T0, and V126-T0—showed *Ppd-B1* expression patterns that were similar to each other, with peaks shifted to ZT8 and low expression during the night. All these samples shared the same mutation type and location: a 1 bp insertion downstream of the CHE binding site. Additionally, V126-T0 had a 1 bp deletion downstream of the CHE binding site.

In V8-T0, *Ppd-B1* contained an 11 bp deletion that removed the CHE binding site entirely, resulting in the weakest *Ppd-B1* expression and a shifted expression pattern.

**Fig. 3:**
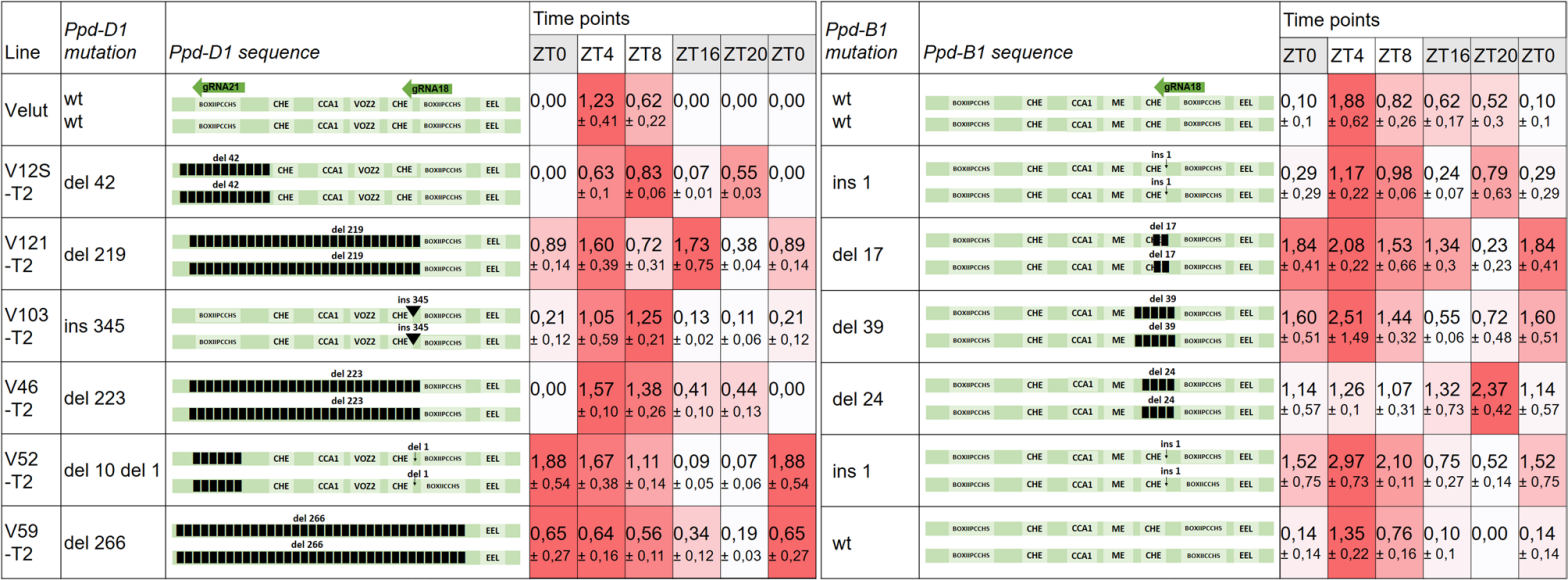
Description of mutations and the diurnal expression patterns of *Ppd-D1* and *Ppd-B1* genes in T2 plants. Gene expression levels are shown schematically using a color scale—the darker the color, the higher the level of expression at a given time point. The numbers represent the relative expression levels based on three biological replicates, with ± indicating the standard error of the mean. TFBSs for CHE (CCA1 HIKING EXPEDITION), CCA1 (Circadian Clock Associated 1), ME (morning element), EEL (evening element) are indicated.

The diurnal expression analysis was repeated using T2 lines carrying homozygous mutations (Fig. 3, Table S7). Not all lines analyzed at the T0 generation were selected for this subsequent expression study, as homozygous mutant lines could not be obtained for some, while others did not produce viable seeds. Heading time was assessed for all T2 lines included in the expression analysis.

Wild-type allele expression patterns aligned with expectations, peaking 4 h after dawn and exhibiting significantly reduced or absent expression during night-time points (ZT0, ZT16, and ZT20). This pattern was also characteristic of the *Ppd-B1* gene in line V59-T2, which lacked mutations. In contrast, all mutant lines displayed altered expression patterns for the *Ppd-D1* and *Ppd-B1* genes compared to the wild-type allele.

Strong pre-dawn expression (ZT0) was observed for the *Ppd-D1* gene in lines V52-T2 (10 bp and 1 bp deletions affecting the BOXIIPCCHS region) and V59-T2 (266 bp deletion removing the entire core region), as well as for the *Ppd-B1* gene in lines V121-T2 (17 bp deletion removing half of the second CHE binding site) and V103-T2 (39 bp deletion completely removing the second CHE binding site).

Interestingly, expression of the *Ppd-D1* gene in line V46-T2 (223 bp deletion removing most core region elements) resembled the normal pattern, showing no expression at ZT0 and reduced expression during nighttime points; however, expression remained consistently detectable at ZT16 and ZT20. Expression of *Ppd-D1* in line V12S-T2 (42 bp deletion removing BOXIIPCCHS) exhibited a bimodal pattern, peaking during the day (ZT8) and again in the middle of the night (ZT20).

The *Ppd-B1* gene in lines with small sequence changes, such as a single base pair insertion adjacent to the CHE binding site in lines V12S-T2 and V52-T2, was expressed during nighttime points.

Comparisons between T0 and T2 plant expression patterns were possible for several lines. For example, *Ppd-D1* expression in V59-T0 (heterozygous 266 bp deletion/wt) was similar to that in the V59-T2 line (homozygous 266 bp deletion), displaying relatively constant expression across all time points with reduced expression during one nighttime interval. The *Ppd-D1* expression pattern in V103-T0 (heterozygous insertion of 345 bp/deletion of 4 bp and 1 bp) and the V103-T2 line (homozygous 345 bp insertion) peaked at ZT8, with lower expression at other time points. The *Ppd-D1* expression pattern differed somewhat between V121-T0 (heterozygous 219 bp deletion/wt) and V121-T2 (homozygous 219 bp deletion); however, both exhibited strong nighttime expression and detectable expression across all tested time points.

### Phenotyping of plants with different mutations in the *Ppd-1* promoter regions

**Fig. 4:**
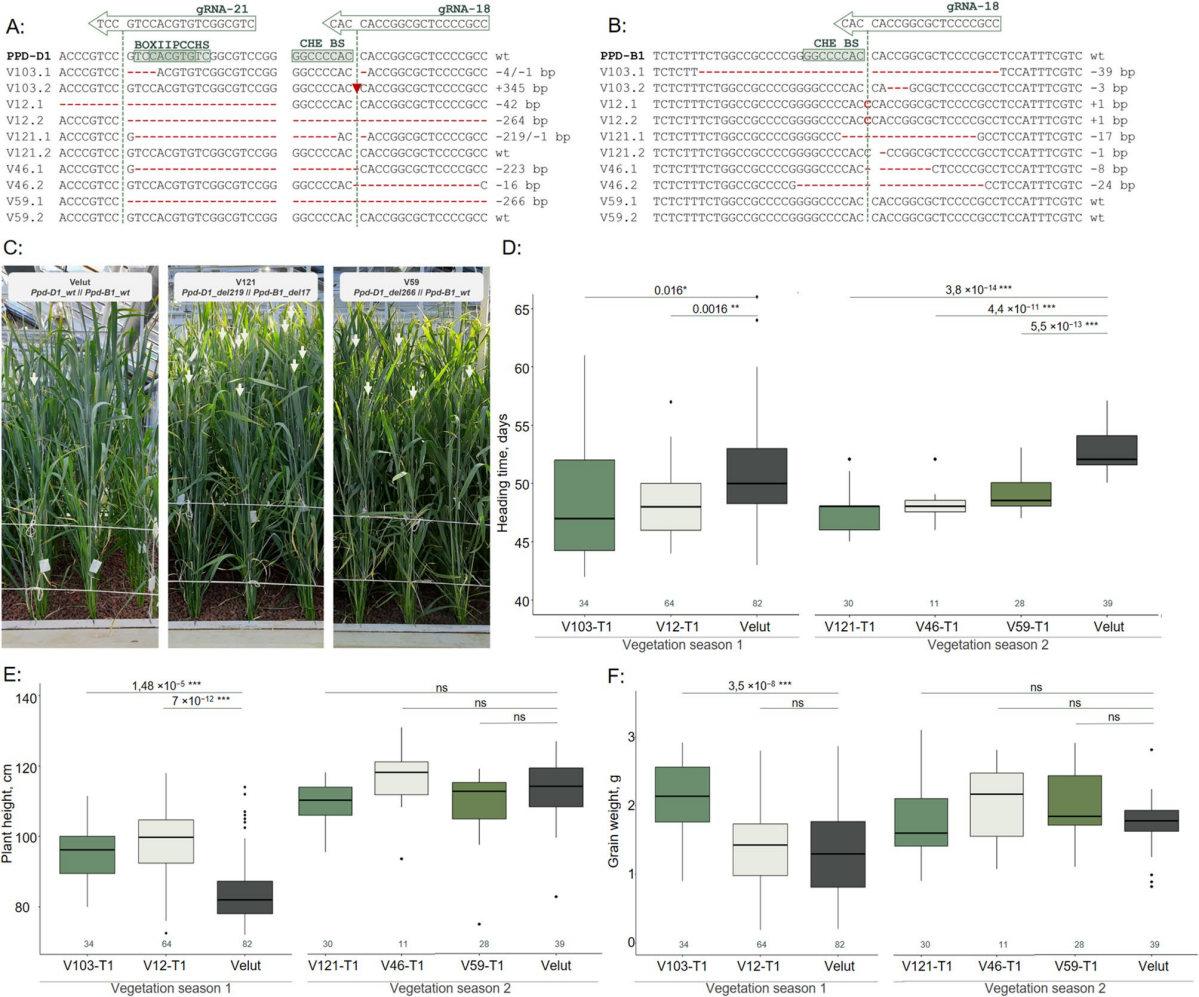
Description of T1 plants. Mutations in promoter region of *Ppd-D1* (**A**) and *Ppd-B1* (**B**), green arrows represents gRNA position, green dashed line indicates site of hypothetical site for digestion by Cas9, green boxes of wt sequence indicate potential TFBS, left column—name of the plant, right column—description of the mutation, mutations in the sequence are colored in red; **C** photo of T1 plant families in a greenhouse, light-green arrows point out wheat ears; **D** boxplot for heading time, **E** plant height, and **F** grain weight of T1 plant families. The dark-green numbers below each box indicate the number of analyzed plants. Statistical significance (*p* values) between groups is shown above the boxes. Groups of plants are outlined according to the vegetation season in which they were grown. Solid dots above and below the boxes represent outliers. Identical line numbers in T1 and T2 indicate a common origin from the corresponding T0 mutant.

We evaluated the heading time of T1 and T2 plants with different mutations. The selected T1 plants were grown under controlled long-day conditions in a greenhouse. We conducted two vegetation seasons to assess heading time (HT) in the T1 plants, with wild-type plants used as controls in each season. For this experiment, we selected five samples with different types of mutations, primarily deletions of varying lengths (Fig. 4). Information about the segregation of plasmid insertion and mutations is provided in Table S4. V46-T1 and V121-T1 were plasmid-free, while the other samples showed segregation of the plasmid insert close to a 1:1 ratio.

ANOVA indicated a significant difference in heading time among the different T1 plants compared to the wild-type Velut plants (Fig. 4, Table S5). All analyzed plants with mutations—V103-T1, V12-T1, V121-T1, V46-T1, and V59-T1—showed reduced heading times. The difference in HT ranged from 2.1 to 5.4 days.

In the V103 plants, all T1 progeny carried mutations in the *Ppd-D1* promoter, including either a 345 bp insertion, a combination of 4 bp and 1 bp deletions, or heterozygous states between these mutations. In the *Ppd-B1* promoter, a 39 bp deletion was detected, either in the homozygous state or in combination with a 3 bp deletion in heterozygotes. Although these groups did not differ significantly in heading time, they initiated heading 2.6 days earlier on average than the control plants.

The V12-T1 plants were divided into three haplotypes, all of which carried a 1 bp insertion in the *Ppd-B1* promoter. These were combined with either a 264 bp deletion, a 42 bp deletion, or a heterozygous state between these two mutations in the *Ppd-D1* promoter. While these haplotypes did not differ from each other in heading time, they also headed earlier than the wild-type plants by an average of 2.5 days.

V121-T1 exhibited the earliest heading time (HT), with plants in this family heading approximately 5.1 days earlier than Velut. Among the different mutation groups, the combination of a 219 bp deletion in *Ppd-D1* and a 17 bp deletion in *Ppd-B1* resulted in the earliest phenotype, with heading occurring about 6 days earlier.

V46-T1 headed 4.5 days earlier than Velut, with plants in this family showing a 223 bp deletion in the *Ppd-D1* promoter, either in the homozygous state or as a heterozygote with the wild-type allele. Additionally, a heterozygous 24 bp deletion in the *Ppd-B1* promoter was also present.

V59-T1 did not exhibit any segregation, with all plants carrying a heterozygous 266 bp deletion in *Ppd-D1* and the wild-type *Ppd-B1* allele. These plants headed 3.7 days earlier than Velut.

We also assessed plant height and grain weight (for the main spike). V103-T1 and V12-T1 were significantly taller than Velut (Fig. 4E), while the other T1 families, including V121-T1, V46-T1, and V59-T1, showed no significant height differences. Additionally, V103-T1 had a higher grain weight compared to Velut, while the other plants did not show significant distinctions (Fig. 4F).

**Fig. 5:**
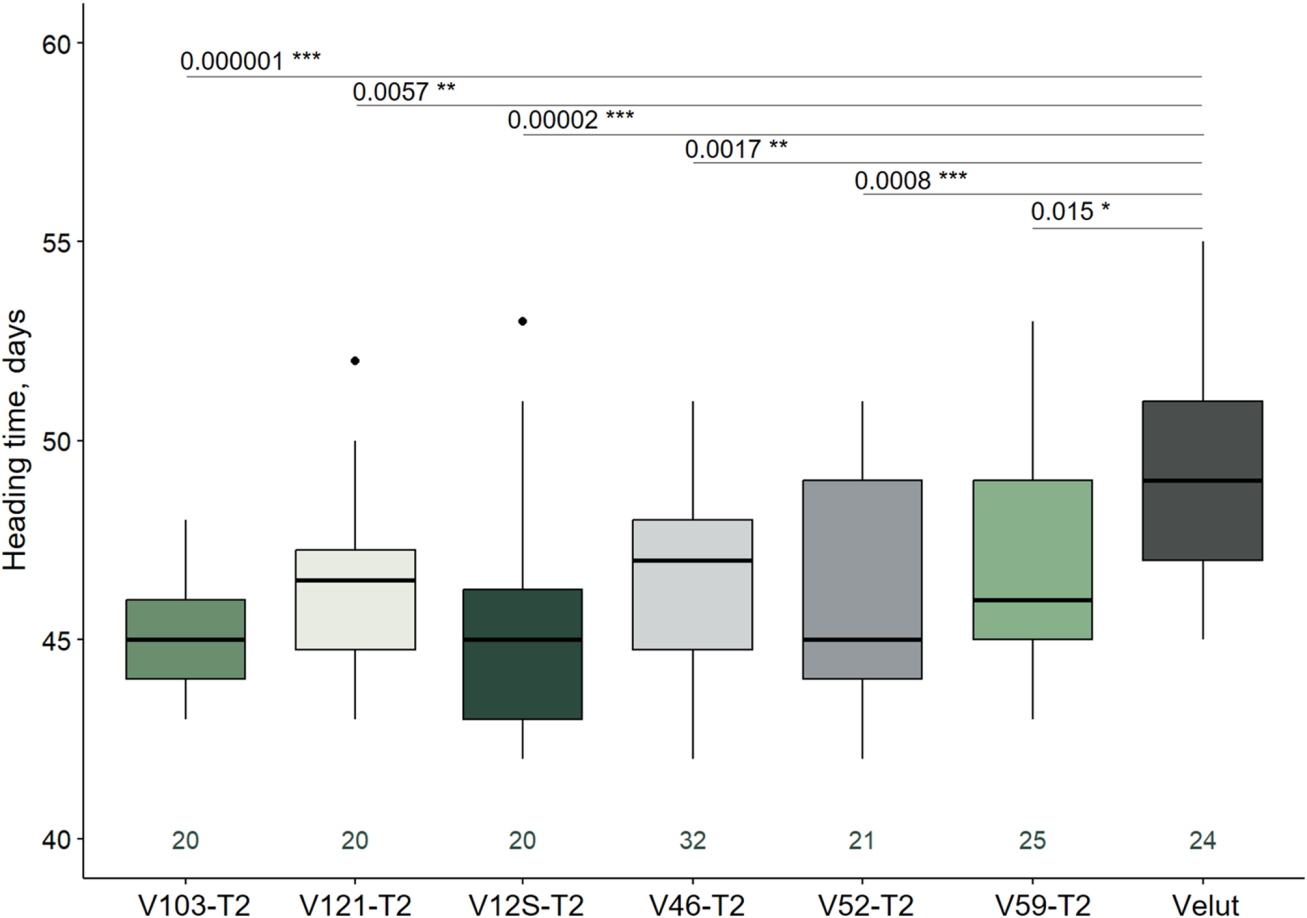
Boxplot for heading time of T2 plants with homozygous target mutations. The dark-green numbers below each box indicate the number of analyzed plants. Statistical significance (p values) between groups is shown above the boxes. Groups of plants are outlined according to the vegetation season in which they were grown. Solid dots above and below the boxes represent outliers. Identical line numbers in T1 and T2 indicate a common origin from the corresponding T0 mutant.

Evaluation of heading time in T2 lines with homozygous mutations in target genes under experimental field conditions (long-day photoperiod ranging from 15 h of daylight in May to 17.5 h in June) showed that all lines headed significantly earlier than Velut (Fig. 5, Table S6). Line V103-T2, carrying a 345 bp insertion in the *Ppd-D1* promoter combined with a 39 bp deletion in the *Ppd-B1* promoter, headed the earliest—4.5 days earlier than Velut. V103-T1 plants with the same mutation combination headed 4.4 days earlier, demonstrating comparable results.

Line V12S-T2, with a 42 bp deletion in the *Ppd-D1* promoter and a 1 bp insertion in *Ppd-B1*, also headed early—4 days earlier than Velut, faster than T1 plants with the same mutations, which headed 2.5 days earlier than the control.

Lines V121-T2 (219 bp deletion in *Ppd-D1* promoter and 17 bp deletion in *Ppd-B1*), V46-T2 (223 bp deletion in *Ppd-D1* promoter and 24 bp deletion in *Ppd-B1*), and V59-T2 (266 bp deletion in *Ppd-D1* promoter) headed 3, 2.8, and 2.5 days earlier than Velut, respectively. A similar trend was observed for T1 plants carrying the same mutations, which headed 4.9, 4.5, and 3.7 days earlier than Velut, respectively. However, it should be noted that heading time assessments for T1 and T2 plants were conducted under different conditions—greenhouse and experimental field, respectively—and environmental factors may also influence heading time.

## Discussion

Most studies on plant gene editing have focused on generating mutations in a single specific gene, with target sites typically chosen within coding regions. This strategy usually results in complete gene knockouts. However, for many genes such an approach is not effective due to their essential biological functions; loss of function can lead to reduced fertility, lethality, or severe phenotypic alterations. Consequently, these mutations often have limited scientific and breeding value. For example, Errum et al. (2023) reported induced mutations in the exons of *Ppd-1* genes that disrupted gene function and resulted in delayed flowering^26^—an undesirable outcome for wheat cultivation in Western Siberia.

Recent advances now enable fine-tuning of gene expression through editing of regulatory elements in untranslated regions, promoters, or enhancers. Such “non-silent” editing events allow precise regulation of target gene expression using CRISPR–Cas technologies^1,27,28^. These strategies can improve key agronomic traits such as flowering time and plant architecture without negatively affecting other characteristics, offering a powerful approach for crop improvement.

For instance, Li et al. (2019) edited the promoter of the *Xa13* gene, which confers susceptibility to bacterial blight in rice^29^. Although complete knockout of *Xa13* enhanced resistance, it also impaired important agronomic traits and caused sterility because *Xa13* plays a crucial role in pollen development. In contrast, deletion of a specific promoter region associated with *Xanthomonas oryzae* susceptibility generated *Xa13* alleles with practical breeding value, without undesirable pleiotropic effects.

Promoter editing has also been successfully used in other crops: to create citrus varieties resistant to citrus canker (*CsLOB1*)^30^; to develop rice with broad-spectrum resistance to bacterial blight (*OsSWEETs*)^31^ or altered starch composition (*OsGBSSI*)^32^; and to increase rice yield by resolving the tradeoff between grains per panicle and tiller number through promoter editing of *IPA1* (*IPA1-Pro10*)^33^. Similarly, CRISPR–Cas9 has been applied to edit the promoter of *ZmCLE7*, a component of the CLAVATA-WUSCHEL pathway controlling meristem development. A homozygous null allele of *ZmCLE7* causes fasciated and disorganized ears with reduced yield, whereas promoter-edited *ZmCLE7* alleles preserved normal ear architecture and substantially increased yield^34^. These studies revealed a novel CLE signaling pathway and generated new alleles for maize improvement^34–36^.

Together, these studies demonstrate that targeted promoter modifications provide a reliable and versatile approach for fine-tuning gene expression and improving yield-related traits. In a similar way, the collection of lines generated in our study can serve not only as models for investigating the regulatory dynamics of *Ppd-1* genes, but also as a source of novel alleles for breeding. These lines are of particular value because they accelerate heading time without compromising yield. We demonstrate here that targeted promoter editing offers a rapid method to generate early-heading wheat lines, whereas introgression of naturally occurring *Ppd-1* promoter alleles through traditional breeding remains labor- and time-intensive.

### Transformation and regeneration in a non-model wheat variety

Genotype-dependent transformation and regeneration remain challenges in cereal genome editing, including for bread wheat. For this study, we selected a genotype known for its high in vitro regeneration capacity^37^. Regeneration response and transgene performance following biolistic gene transfer depend on various parameters, such as particle type, size, quantity, and acceleration; DNA amount and structure during particle coating; and tissue type^38,39^.

In most studies on genome editing in common wheat, immature embryos are used as explants^22^. However, some studies have shown that using callus can significantly enhance both transformation efficiency and plant regeneration. For instance, while the Chinese Spring (CS) variety is generally considered highly efficient in regeneration^40^, Michard et al. reported that CS behaves as a recalcitrant genotype for biolistic transformation. In their experiment, more than 3000 immature embryos were bombarded, but no transformed plants were obtained^41^. In contrast, Miroshnichenko et al. successfully generated 23 independent T0 transgenic plants from 603 explants by using immature callus of CS as explants^42^. The use of immature callus as explants has also enabled successful transformation in wild relatives of common wheat, including *Triticum dicoccum* and *Triticum timopheevii*^43^.

Among the factors influencing the efficiency of stable transformation are the selectable marker gene, the selection strategy, and the genotype’s sensitivity to the selection agent. The *bar* gene, which confers resistance to the herbicide phosphinothricin, is widely used as a selectable marker for cereal transformation, including wheat^44^. However, a significant drawback of using the *bar* gene is high frequency of escape plants (non-transformed plants that survive in vitro selection), which has commonly been reported.

For example, in the study by Witrzens et al.^45^, out of 92 wheat regenerants, the majority—87 plants (95%)—did not contain the vector insert. Similarly, Nehra et al.^46^ reported that 50% of regenerated plants (8 out of 16) were escape plants. Gadaleta et al.^47^ transformed durum wheat and found that the selection efficiency, calculated as the number of transgenic plants divided by the total number of plants, was only 26.4%. High escape frequencies have also been reported in other cereal transformation studies^48,49^.

In our experiment, most regenerated plants (99 out of 133) from *in vitro* culture did not contain the vector insert. However, only seedlings that survived and developed into fully established plants with robust root systems on selective medium containing phosphinothricin were included in the analysis.

This observation suggests that the Velut line may possess a natural tolerance to phosphinothricin. Indeed, the literature reports natural herbicide resistance in certain cultivated plant species, such as barley^50^, corn^51,52^, alfalfa^53^, soybeans^54,55^, and sugarcane^56^. For future studies, we may need to modify the selection strategy for the Velut wheat line. To suppress the growth of non-transgenic plants, we recommend increasing the concentration of phosphinothricin in the induction medium (WCIM, Wheat callus induction medium). Alternatively, the step involving a one-week rest on WCIM medium without the selection agent immediately following bioballistics could be omitted. An experiment on the genetic transformation of a recalcitrant tomato variety demonstrated that adding a non-selection stage to the protocol increased the number of shoots; however, most of these shoots were non-transgenic^57^. Another option could involve adopting a different resistance gene and selection agent, such as the *hpt* gene (encoding hygromycin phosphotransferase), which provides resistance to the hygromycin B, to improve the protocol’s efficiency.

In our experiment, the plantlet regeneration rate, calculated as the ratio of regenerated plants to the total number of immature callus subjected to transformation, was 14.3%. A total of 133 plants were regenerated on selective medium containing phosphinothricin. Among these, 35 plants (26.3% of all regenerated plantlets) harbored the plasmid, with 9 of them containing the vector insertion but showing no mutations. Consequently, the stable transformation frequency was 3.76%, which is comparable with previously reported data for wheat^58–60^.

Since the biolistic method was used for plasmid delivery, we hypothesized that some of the 99 plants without vector DNA insertions might harbor mutations at the target loci. This hypothesis is supported by previous studies demonstrating that plant genome editing can occur without integration of DNA editing system components into the genome^61^. Therefore, we decided to analyze all 133 obtained plants, not just the transgenic ones, for the presence of mutations in the target regions. One of the aims of our study was to evaluate the potential for selecting non-transgenic plants with target mutations from the T0 plant population generated using a conventional single-vector biolistic transformation method. Genome editing in wheat, as in other species, has been previously achieved through the transient expression of a DNA vector^62–65^. Little is known about the mechanism of plasmid DNA integration following biolistic transformation, but the number of transgene copies can range from zero to several dozen, depending on the amount of DNA delivered to the cells^66–68^. In our experiment, 46 plants (34.6% of the total regenerated plants) were edited, with 20 of these being plasmid-free. Notably, nearly half of the plants with mutations lacked plasmid insertions, indicating that editing likely occurred through transient vector expression. If we had limited our analysis to only transgenic plants, the editing rate in this experiment would have been 2.8%.

When comparing the mutations detected in plants with and without vector insertions, we observed no notable differences. Both groups included short and long indels, either in homozygous or heterozygous states, paired with a different mutation or with the wild type, in both the D and B genomes (Table S3). Additionally, probable chimeric plants were identified in both groups. These results confirm the possibility of effectively selecting target, non-transgenic mutants in the T0 generation.

One limitation to the widespread application of the CRISPR/Cas9 genome editing method is the complexity and labor-intensive process required to eliminate transgenic vector inserts from target mutant plants^23,69^. In cereals, a thorough molecular analysis of individual seedlings, which increases in number with each generation, is necessary to detect and remove transgenic inserts from the plant genome.

Continuous expression of CRISPR/Cas9 editing genes within the genome can lead to undesirable effects, particularly in subsequent generations, including off-target edits and tissue mosaicism. This is due to ongoing editing by the CRISPR enzyme and guide RNA, which can target both intended and unintended sequences^70,71^. Some studies have shown that transgene-free edited plants can be obtained in the T0 generation using RNP complexes; however, this approach remains challenging as it requires significant effort to identify edited plants^72,73^.

The transient expression for genome editing offers a more efficient approach for obtaining genome-edited plants free from vector insertions. This method saves time, labor, and cost, as it requires only the transient expression of the vector in cells with strong regenerative capacity. This approach has been developed for *Agrobacterium*-mediated transformation^64,74^ and is also well suited to the biolistic transformation method^65,75^.

### Development of Velut wheat line with accelerated heading

Each year, additional genes and alleles influencing wheat heading time are identified. Recent discoveries include genes such as *TaELF3*, *TaLUX* (*WPCL*), *WAPO1*, *TaZIM*, and various miRNAs^76–80^. However, the genes most commonly used in breeding to modify wheat heading time—such as *VRN-1*, *VRN-3*, and *PPD-1*—were identified earlier^4,81,82^.

These genes play key roles in the regulatory pathways that control heading time and show extensive allelic diversity. This diversity enables breeders to select specific alleles from donor varieties to introgress into target genotypes. However, a significant challenge in using these genes as genome editing targets to accelerate heading time is that their effects are generally associated with mutations in regulatory regions rather than gene knockouts. Such mutations lead to changes in gene expression patterns. While a few studies have focused on editing these key genes, most have targeted gene knockouts rather than modifications in their regulatory elements.

For example, *TaFT-D1* has been successfully targeted for genome editing, where mutations in the first exon—specifically deletions of 1, 2, or 7 bp – led to a 2–3 day delay in heading^83^. Similarly, in a study by Errum et al., mutations introduced into the coding regions of *Ppd-A1*, *Ppd-B1*, and *Ppd-D1* genes altered spike architecture and delayed flowering. The authors also noted significant effects of these mutations on plant growth and productivity^26^.

In contrast, other studies have aimed to accelerate wheat heading time by editing the *VRN1* gene. One study targeted the promoter region of *Vrn-A1* and generated plants with various mutations, where 1–4 nucleotide insertions/deletions had no effect on heading time. However, an 8 bp deletion in this region accelerated head emergence by up to 3 days^42^.

In our study, we successfully generated common wheat plants with various mutations in the regulatory regions of the *Ppd-D1* and *Ppd-B1* genes through genome editing, resulting in accelerated heading by up to 5.1 days under long-day conditions. Flowering time was assessed under long-day conditions to reflect the native growing environment of the Velut, which exhibits delayed heading in such settings. Although photoperiod-insensitive alleles of *Ppd-1* are known to have more pronounced effects under short days, previous studies have shown they can also accelerate flowering under long-day conditions^84,85^.

All haplotypes with mutations in the target genes differed significantly from the control, yet they showed no significant differences among themselves. Unfortunately, the presence of mutations in two homoeologous target sequences complicates the precise assessment of the individual effects of each mutation on the phenotype. When plants segregate with a single mutation in *Ppd-D1*, an additional mutation in *Ppd-B1* is frequently present in the background. By comparing haplotypes V121-T1 and V59-T1—both carrying long deletions, but with V59-T1 lacking mutations in *Ppd-B1*—we suggest that the combination of mutations in both *Ppd-D1* and *Ppd-B1* may contribute to the observed acceleration in heading time.

Assessment of heading time for T2 lines carrying homozygous mutations in the target genes under field conditions revealed that every line initiated heading significantly earlier (2.8–4.5 days) than Velut.

*PPD-1* genes are known to have pleiotropic effects on various agriculturally important traits. Therefore, we assessed the effects of mutations in the T1 generation on plant height and grain weight per spike (GWPS). Both, V12-T1 and V103-T1 family plants were higher than Velut and V103-T1 has larger GWPS. Interestingly, only plants from the V103-T1 family showed a significant difference in GWPS compared to Velut, as this family was the only one in the sample carrying an insertion, rather than a deletion, in the target region of the *Ppd-D1* gene.

In future studies and selection of wheat lines, considerable attention will be given to the height and yield characteristics of plants with different mutations types. The increased height observed in V12-T1 and V103-T1 plants is an undesirable trait, as these plants may be prone to lodging when grown in the field^86^.

The non-Mendelian segregation ratios observed in the T1 population derived from the selfing of T0 plants are frequently reported in the literature. One possible explanation is the limited number of T1 seeds produced by T0 plants, which may not provide sufficient representation of Mendelian inheritance patterns^42^. Additionally, segregation deviations, such as 1:1 or 1:2 ratios, could result from irregularities in gamete or seed formation, as has been previously suggested^87,88^.

### Regulation of *Ppd-1*

Based on literature data regarding known transcription factor binding site motifs that may be involved in regulating *PPD-1* gene expression, we mapped these sites onto the *Ppd-D1* and *Ppd-B1* promoter sequences (Fig. 1D).

First of all, both genes contain two binding sites for CHE, one for CCA1/GATA and LUX, additionally there were three BOXIIPCCHS (ACGTGKM), two of which occur in gRNA21 sequence. Further, in promoters of *Ppd-D1* and *Ppd-B1* there are two complex binding sites—Evening Element-like motif (EEL) AATATCT and Morning element (ME)—CCACAC (Fig. 1D). Interestingly, *Ppd-B1* has an additional ME position, just before the second CHE binding site. In this region, *Ppd-D1* identifies TFBSs (transcription factor binding sites) for VOZ2 and FAR1. Thus, it is interesting to note that although most of the TFBSs in the significant part of the *Ppd-D1* and *Ppd-B1* promoters are the same, there are some differences.

PhyC and PhyB are considered key regulators of photoperiod sensitivity and the transition to flowering in wheat and barley^13,14,89,90^. These genes have been shown to regulate *PPD-1* expression^13,14^. Later, the same authors demonstrated that the regulation of *PPD-1* by phytochromes is likely mediated by the evening circadian rhythm complex protein EARLY FLOWERING 3 (ELF3)^15^. ELF3 indirectly binds to the *PPD-1* promoter through LUX ARRHYTHMO (LUX), another component of the “evening complex”. LUX binds to GATWCG motifs, which are found in the *PPD-1* promoter (Fig. 1D). However, in our collection of plants with deletions in promoter of *PPD-1*, most plants contained indels that did not disrupt the LUX binding site (and, consequently, ELF3), nor the EEL, a complex regulatory element that integrates binding sites for several “evening” regulatory factors. These plants exhibited dysregulated expression patterns of the *PPD-1* genes and had earlier heading time, even under long-day conditions.

Here, we demonstrated that plants with deleted CHE binding sites headed earlier and expression patterns of upstream *PPD-1* gene was changed as compared to Velut plants. In earlier studies, the CHE binding site was identified as the primary putative cis-regulatory element within the *PPD-1* promoter^17^. In confirmation of which, Wei et al. (2021), using GUS gene expression under different *Ppd-D1* promoter variants in a transgenic *Arabidopsis* system, concluded that the CHE binding sites are core regulatory elements responsible for the high-level expression of *Ppd-D1b* three hours after dawn. It can be supposed that the regulation of *PPD-1* gene expression is highly complex, with different regulators acting at distinct times of the day^19^.

It is also noteworthy that even minor changes in the promoter regions of the *Ppd-1* genes, such as the insertion of a single nucleotide immediately downstream of the CHE binding site (as observed in the *Ppd-B1* promoter of lines V12S-T2 and V52-T2), resulted in altered expression patterns. This suggests that not only the transcription factor binding site itself but also its adjacent sequences are functionally significant.

Moreover, based on the expression patterns observed in lines with mutations that specifically deleted the BOXIIPCCHS element without affecting CHE binding sites, it can be inferred that these cis-elements are also crucial regulators of gene expression, as their removal (as in line V12S-T2) similarly altered expression patterns.Based on the observed expression patterns, we hypothesize that CHE functions as a repressor, and that additional regulatory elements, beyond CHE and the previously described ELF3 and LUX, are also involved in the regulation of *PPD-1* expression. Thus, the regulation of wheat *PPD-1* genes is likely achieved through coordinated interactions among multiple transcription factors. This complex interplay ensures precise temporal control of gene expression, which is critical for photoperiod sensitivity and heading time regulation. In this study, we generated a collection of wheat lines carrying different promoter mutations in *Ppd-D1* and *Ppd-B1*, which provide valuable material for future investigations of the regulatory mechanisms controlling expression of these genes.

## Materials and methods

### Plant material for tissue culture experiments

The experiments were performed using a spring common wheat line Velut (obtained from the collection of the Department at the Plant Genetics of Institute of Cytology and Genetics SB RAS). Detailed pedigree of this line presented in Berezhnaya et al.^91^. This genotype carried *Ppd-D1b*, *Vrn-A1a*, *Vrn-B1c*, *vrn-D1* and *Vrn-B3d* alleles of key heading time genes. The ability to regenerate plants *in vitro* of Velut was shown to be very high (13.7 plantlets per embryogenic explant)^37^.

Donor wheat plants were grown in an environment-controlled climatic chamber (Fitotron SGR 121, Weisstechnik) at 18 °C day/15 °C night with a 12 h photoperiod and 70% humidity for 6 weeks for better tillering and earing synchronization. Then plants were transferred into chamber with 16 h photoperiod (PPFD, photosynthetic photon flux density, approximately 440 µmol m^−2^ s^−1^) at 21 °C day/18°C night and 70% humidity. At the tillering stage (GS20/24 by Zadoks scale), plants were fertilized with Osmocote exact (N, P, K: 15%, 9%, 12%; Scotts, The Netherlands).

### Plant tissue culture

Immature seeds were harvested at 14–16 days post-anthesis. Isolated caryopses were immersed firstly in 70% ethanol (v/v) for 1 min and then in the 50% commercial bleach solution (Domestos) with occasional gentle shaking for 10 min. Surface-sterilized caryopses were rinsed five times with distilled sterilized water. Embryos of about 1–1.5 mm in length and translucent in appearance were isolated from the caryopses. Then, 100–150 isolated embryos per petri dish were placed scutellum side up, with the embryo axis faced down, in contact with the callus induction media (WCIM) described by Sparks and Doherty^92^. In our experiment, WCIM contained 2.5 mg/L Dicamba instead of 2,4-Dichlorophenoxyacetic acid and all other components were the same. Embryos were grown for 5–7 days at 24 °C in the dark to obtain embryogenic callus. At this stage, we selected only responsive embryos with visible callus growth on the scutellum and separated embryo axis from the scutellum with sharp tweezers (Dumont, style 7, cat. № 0203-7-PO). Scutella that were fading to yellow or had a brownish tinge were discarded. Selected immature embryo-derived (IE) callus (or shortly «immature callus») were maintained on WCIM in the dark in the same orientation for one more day before bombardment. For 4–6 h prior to bombardment and 16 h after bombardment IE-calluses were cultured on the osmotic media containing 0.4 M mannitol, 4.3 g/l MS basal salt with vitamins, 30 g/l maltose, 2.5 mg/l Dicamba in the dark^43^. Embryo-derived callus were located within the central 2 cm diameter circular area of a petri dish. Each dish contained 30–35 explants.

All steps of post-transformation culture, selection, and plant regeneration were performed as previously described^92^. Following bombardment, IE-callus (15 per plate) uses in the same orientation were placed onto fresh WCIM medium without selection agent and containing 2.5 mg Picloram mg/l and incubated at + 24 °C in the dark for one week. After, 10 IE-callus were moved to the first selection medium (WP5) with 5 mg Phosphinothricin (PPT) and incubated at 24 °C in the dark for 2 weeks. After 2 weeks enlarged callus were transferred to second selection medium with 10 mg PPT (WP10) for 3 weeks at 24 °C in the dark. After the callus were transferred to regeneration medium (WRZP5) with 5 mg PPT under fluorescent lights (~ 100 µmol m^−2^ s^−1^) at 24 °C with a 12-h photoperiod for 2–3 weeks. Appeared small green plantlets were transferred to the rooting medium WRP5 with 5 mg PPT in plant culture containers (Phytohealth, SPL). Regenerated strong plantlets T0 with established root system were moved to soil, analyzed and grown to maturity for obtaining T1 seeds.

### Target locus selection and sequencing

Genomic DNA was extracted from 3 to 4 seedlings following a modified CTAB protocol^93^. The fragment of the promoter region of the *Ppd-D1* and *Ppd-B1* genes was amplified from the genomic DNA of Velut using the primers PpdD1_pr_F1/PpdD1_int1_R1 and F-Ppd-5UTR/R-Ppd-5UTR (Table S1) using the LR HS-PCR (BioLabMix, Russia). PCR products were isolated from the 1% agarose gel using a Zymoclean™ Gel DNA Recovery Kit (ZymoResearch, Irvine, CA, USA) and directly sequenced using a BigDye Terminator v.3.1 kit (Applied Biosystems, Forster City, CA, USA) with flanking and nested primers (Table S1). Sequencing products were analyzed using an ABI 3130XL GeneticAnalyser at the SB RAS Genomics Core Facility. To determine the *Ppd-A1* genotype of the Velut cultivar, we used allele-specific primers targeting the known *Ppd-A1a.1* deletion^17^. The amplification profile matched the wild-type allele, confirming that Velut carries a photoperiod-sensitive *Ppd-A1* allele.

### sgRNA design

We designed gRNAs to target binding sites of CHE transcription factor (which are potential repressors of *PPD-1*) to create deletions (about 200 bp) that would remove both CHE sites. To choose appropriate sequences of gRNAs we used software CRISPR-P 2.0 ^94^, Benchling (https://www.benchling.com/), CRISPRdirect^95^, sgRNA-Designer^96^, wheatCrispr^97^. The gRNAs selected in these programs were compared and only those that, according to the results of several programs, were characterized by high efficiency were selected for further work. Thus, 10 gRNAs were selected (5 for each side). Additionally, specificity was assessed using Blast (Ensemble). The sequences selected for this work are presented in Table S2.

### *In vitro* cleavage assay

Primers (20 µM) for 10 gRNAs with a four-base ACTT overhang at the 5’-end and CAAA overhang at the 3′-end were mixed with Annealing buffer (10mM Tris, pH 7.5–8.0, 50 mM NaCl, 1mM EDTA) in a volume of 10 µl and annealed using the following program: 94 °C for 5 min, followed by gradual cooling to 35 °C (− 1 °C/min). Then annealed primers were ligated in pUC57-sgRNA expression vector linearized with BsaI-HFv2 (NEB, USA). The resulted construction was used for in vitro transcription of sgRNAs with HiScribe T7 Quick High Yield RNA Synthesis Kit (New England Biolabs) according to the manufacturer’s instructions. 30 nM of synthesized sgRNA and 30 nM of EnGen Spy Cas9 nuclease (NEB, USA) were mixed in a volume of 30 µL and incubated for 10 min at room temperature to form the RNP complexes. Then 3 nM of the PCR-product of *Ppd-D1* target region was added and incubated at 37 °C in NEB reaction buffer for 2 h. The digestion products were separated on a 2% agarose gel. Densitometric analysis compared the luminosity of restriction products with an intact control was performed to comparatively assess the activity of RNA guides. The ImageJ 1.53 (Windows version of NIH Image, https://imagej.net/ij/), program converts the glow of bands in an agarose gel into graphs^98^. Each band has its own peak, and the area under the peak is the intensity of the glow^99^. By comparing the areas under the peaks of control and experimental samples, we can say what percentage of DNA was subject to restriction. The gRNA activity is equal to the percentage of the luminosity of the restriction products relative to the luminosity of the intact fragment.

### *In vivo* gRNA efficiency analysis

The protoplasts were extracted from the 2-week old wheat seedlings following the protocol published by Brandt et al.^100^ with minor modifications. RNP complexes with tested sgRNAs were assembled from 20 µg of the EnGen Spy Cas9 NLS (NEB, USA) and 20 µg of the sgRNA. Protoplasts (5 × 10^5^) mixed with RNP were transformed using the 40% PEG 6000 solution^101^. After 2 days, DNA from the protoplast was extracted, promotor region of the *Ppd-D1* gene was amplified with PpdD1_prot_F3 and PpdD1_prot_R3 (Table S1) and gRNA efficiency was analyzed with the GeneArt™ Genomic Cleavage Detection Kit (Invitrogen, USA) according to the manufacturer’s protocol. The amplicons of *Ppd-D1* were subsequently sequenced and sequences were analyzed with DECODR 3.0 ^102^.

### Construction of plasmid for gene editing

To create plasmid for the biobalistic transformation of wheat we used plasmids from MoClo Kit: pFH85 (Addgene plasmid #128198; https://www.addgene.org/128198/), pFH33 (Addgene plasmid #128198), pICH47751 (Addgene plasmid #48002), pICH47761 (Addgene plasmid #48003), pFH66 (Addgene plasmid #131765) pFH114 (Addgene plasmid #128408), pICSL4723-P1 (Addgene plasmid #86173)^103,104^. The scheme of the plasmid construction is presented in Fig. S1. They were assembled using the BsaI-HFv2 (NEB, USA) and BstV2I (SybEnzyme, Russia) restriction enzymes and T4 DNA ligase following the protocols described in Hahn et al.^103^.

The resulted plasmid “L2_41780_Ppd_gRNA18 + 21_Cas9_bar” harbored two gRNA with classic sgRNA backbone for SpCas9, each driven by the wheat U3 promoter, wheat codon optimized SpCas9 driven by the maize Ubi1 promoter, and BAR gene (phosphinothricin acetyltransferase) BAR: NOSt driven by the maize Ubi1 promoter (Fig. S2).

### Biolistic transformation

Particle bombardment was performed using the protocol published by Svitashev et al. ^74^ with modifications. Per ten gunshots 50 µl of 40 mg/ml washed 0.6 μm gold particles (Bio-Rad, USA Cat. № 1652262) (prepared with standard protocol) were coated with 900 ng of L2_41780_Ppd_gRNA18 + 21_Cas9_bar, mixed with 100 ng of 35 S-eGFP-nosT using 5 µl (0.5 µl per shot) water soluble cationic lipid TransIT-2020 solution (Mirus, USA). Concentration of the TransIT-2020 was selected according to the^105^ recommendations. The mixture was gently mixed and incubated on ice for 10 min, centrifuged at 8000*g* for 30 s and supernatant was removed. 100 µl of water (10 µl per shot) was added to the pellet and gently resuspended with short sonication. This solution was applied to the macrocarriers set in its holder with small drops and allowed to air dry for approximately 60 min.

Immature callus of wheat were bombarded using a PDS-1000/He Gun (Bio-Rad, USA) with a distance between target cell and stopping screen of 6.0 cm at a helium pressure adjusted for 900 psi rupture disks (200 psi over desired rupture pressure) (Cat. no. 1652328, Bio-Rad, USA) and ~ 28″ Hg of vacuum.

### Identification of mutations and vector integration in regenerated plants

Mutant screening and identification were performed using DNA extracted from the regenerated plantlets, individual shoots of the plant at the heading stage, and T1 plants. The identification of vector integration in the genome was accomplished through PCR with primers targeting vector sequences to *BAR* gene and gRNAs sequences (Table S1). Plants with no PCR amplification from either the sgRNA or *BAR* gene primer sets were considered most likely to be plasmid-free. While no cases of partial integration (amplification from only one primer set) were observed, we acknowledge that this method may not detect very small or fragmented vector insertions.

The presence of mutations in the *Ppd-D1* and *Ppd-B1* genes in the T0 plantlets were identified by PCR screening, followed by Sanger sequencing and next-generation sequencing (NGS) analysis. Primers for identification of mutations in target genes (Table S1) were designed to amplify both short and long regions of target genes’ promoters. PCR amplification was used to identify large indels. These fragments were subsequently recovered from 2% agarose gel using the Zymoclean Gel DNA Recovery Kit (Zymo Research, USA), purified using the ZR DNA Sequencing Clean-Up Kit (Zymo Research, USA), and sequenced. Sequencing products were analyzed using an ABI 3130XL GeneticAnalyser at the SB RAS Genomics Core Facility. For the NGS analysis, primers mPpdD-F4/ mPpdD-R4 and mPpdB-F2/ mPpdB-R2 were used, and the products were purified using the CleanMag DNA PCR magnetic beads solution (Evrogen, Russia), which were sequenced on an Illumina MiSeq platform generating paired-end 250-bp reads. Alignment of FASTQ files and quantification of mutations was performed via CRISPResso2 (https://github.com/pinellolab/CRISPResso2)^106^. Indel frequencies were determined in Cas9 mode with standard parameters, including a minimum homology threshold for alignment to an amplicon set at greater than 60% and an editing window of ± 1 bp around the predicted Cas9 cleavage site. Mutations were considered heterozygous when approximately 50% of the sequencing reads corresponded to modified sequences and 50% to unmodified sequences. To validate these findings, we confirmed the mutations through Sanger sequencing. The Sanger sequencing data were analyzed using the DECODR 3.0 tool with standard parameters. The segregating T1 plants were genotyped using the same approach.

To estimate the copy number of the plasmid insertion, we performed qPCR using a relative quantification assay. This assay compared the relative values of the *BAR* and gRNA region amplicons (Table S1) to an amplicon of the three-copy wheat gene *TaCO2*. All reactions were carried out in duplicate for each plant. A total of 30 ng of DNA was used in 20 µL reactions with a QuantStudio™ 5 Real-Time PCR System (Thermo Fisher Scientific, Vilnius, Lithuania) and PowerUp™ SYBR™ Green Master Mix (Thermo Fisher Scientific, Vilnius, Lithuania). To calculate the number of transgene copies, we used the formula: $~Copy~number~=R~ \times ~E_{{ref}}^{{~~C{t_{ref}}}}/E_{{target}}^{{~~C{t_{target}}}}$, where individual PCR efficiency values (*E*) were evaluated using LinReg software^107^, and *R* corresponds to the copy number of the reference gene.

### RNA extraction and qPCR assay

T0 and T2 plants with various types of mutations in the target genes, along with control plants lacking mutations, were grown in 2-l soil pots under short-day conditions (12 h of light) at 22 °C day/17 °C night and 70% humidity in a WiessTechnick climatic chamber until reaching the flag-leaf stage (GS45)^108^. The analyzed T0 plants exhibited normal growth and showed no morphological abnormalities. T2 plants with homozygous target mutations were grown in the same conditions. Each line was sampled in three biological replicates.

To control for potential tissue culture effects, the wild-type control plant used in the expression analysis was regenerated using the same callus induction and transformation protocol as the edited T0 plants. Only plants exhibiting normal development at the flag leaf stage were selected for RNA analysis. The wild-type control plants used for the T2 expression analysis experiment were sown from randomly selected seeds generated from the T0-control plants.

Flag leaf apices were collected at five time points: immediately before the light was turned on (ZT0), after 4 h of light (ZT4), after 8 h of light (ZT8), after 4 h of darkness (ZT16), and after 8 h of darkness (ZT20). Total RNA was extracted using the Plant RNA MiniPrep Kit (Zymo Research, Tustin, CA, USA), followed by DNase treatment with the RNase-Free DNase Set (QIAGEN, Hilden, Germany). The quantity and quality of the RNA were evaluated with a QUBIT 4 fluorometer using the RNA BR and RNA IQ kits (Thermo Fisher Scientific, Vilnius, Lithuania). First-strand cDNA was synthesized from 2 µg of total RNA using the RevertAid First Strand cDNA Synthesis Kit (Thermo Fisher Scientific, Vilnius, Lithuania) according to the manufacturer’s instructions, with random hexamer primers. For subsequent analysis, 2 µl of a 20-fold diluted cDNA solution was used. Quantitative PCR was performed using a QuantStudio™ 5 Real-Time PCR System (Thermo Fisher Scientific, Vilnius, Lithuania) with PowerUp™ SYBR™ Green Master Mix (Thermo Fisher Scientific, Vilnius, Lithuania). All reactions were run in triplicate, and melting curve analysis was performed to verify the specificity of the PCR products.

All primers, used in this analysis are listed in Table S1. The PCR efficiencies were determined using the LinReg software^107^. The target-gene expression was normalized against reference gene *MetAP1* ^109^. The relative expression values were calculated according to the method proposed by Pfaffl^110^.

### Phenotypic evaluation of T1 and T2

Wheat grains of T1 were planted twice in the spring and fall vegetation seasons of 2024 year under the controlled conditions of the greenhouse under the 16-hour daylight conditions at 23 °C day/18 °C night temperature (Center for Collective Use “Laboratory of Artificial Plant Growth”, FIC Institute of Cytology and Genetics). The length of the photoperiod in the greenhouse was equalized and corresponded to the length of the day in the field of Western Siberia using supplementary lighting. Heading time was defined as the number of days from emergence (GS07) to the stage when approximately half of the spike on the main stem had emerged from the flag leaf sheath (GS55). This was recorded individually for each plant.

To evaluate plant height (PH) the length from the surface of the substrate to the peak of the main stem spike at maturity stage. After thrashing and cleaning grain weight per spike (GWPS) was measured for every main spike.

T1 plants were genotyped and individuals with mutations in homozygous state were selected. T2 grains of these plants were sawn on the isolated experimental field of the Federal Research Centre Institute of Cytology and Genetics SB RAS in Novosibirsk in 2025 in a randomized block design in two replicates on rows of 0.5 m wide, 17 grains per row and a between-row spacing of 25 cm. Sowing took place on May 17, harvesting on August 28 in 2025 year. The average daylight hours in Novosibirsk in 2025 were: 16 h in May, 17.2 h in June, 16.6 h in July, and 15.5 h in August. The conditions for the 2025 growing season were favorable and characterized by optimal moisture, but the weather was slightly cooler than the long-term average. Heading time of T2 plants was defined as previously described above.

### Statistical analysis

Descriptive statistics and analysis of variance (ANOVA) with a post hoc Tukey test were calculated using R for comparison of heading time, plant height and grain weight and pairwise t test for comparison of expression level in each time point between every pair if T2 lines.

## Supplementary Information

Below is the link to the electronic supplementary material.

Supplementary Material 1

Supplementary Material 2

Supplementary Material 3

## Data Availability

The datasets generated and/or analysed during the current study are available in the GenBank repository, PV246050 - PV246166.
