# Supplementary material for: Fine tuning wheat heading time through genome editing of transcription factor binding sites in Ppd-1 gene promoter
Source: Sci Rep. 2025 Nov 26;15:42034. doi: 10.1038/s41598-025-25295-8 (PMC12657949; doi:10.1038/s41598-025-25295-8)
Supplement: Supplementary file 1 — Supplementary Material 1 [file 41598_2025_25295_MOESM1_ESM.pdf]

Velut Mutant Lines *Ppd-D1* Short Indel Sequences around gRNA21

```
                                        gRNA21

                                 -947     -937      -927      -917
                              ....|....|....|....|....|....|....|....|.
                                    BOXIIPCCHS
ENA|DQ885766.1                CCGTCC~GTCCACGTGTCGGCGTCCGGTTCGTTCCCGCGGC
v66_g21_PPD-D1                CCGTCC~~~CCACGTGTCGGCGTCCGGTTCGTTCCCGCGGC
v80_g21_PPD-D1_1              CCGTCC~~~CCACGTGTCGGCGTCCGGTTCGTTCCCGCGGC
v3_g21_PPD-D1                 CCGT~~~GTCCACGTGTCGGCGTCCGGTTCGTTCCCGCGGC
v55_g21_PPD-D1                CCGTCC~~~~~ACGTGTCGGCGTCCGGTTCGTTCCCGCGGC
v73_g21_PPD-D1                CCGTCC~~~~~ACGTGTCGGCGTCCGGTTCGTTCCCGCGGC
v163_g21_PPD-D1               CCGT~~~~~CCACGTGTCGGCGTCCGGTTCGTTCCCGCGGC
v164_g21_PPD-D1               CCGT~~~~~CCACGTGTCGGCGTCCGGTTCGTTCCCGCGGC
v165_g21_PPD-D1               CCGT~~~~~CCACGTGTCGGCGTCCGGTTCGTTCCCGCGGC
v52_g21_PPD-D1_1              CCGTCC~~~~~~~~TGTCGGCGTCCGGTTCGTTCCCGCGGC
v52_g21_PPD-D1_2              CCGT~~~~~~~~~~~GTCGGCGTCCGGTTCGTTCCCGCGGC
v37_g21_PPD-D1                CCGTCC~~~~~~~~~GTCGGCGTCCGGTTCGTTCCCGCGGC
v21_g21_PPD-D1                CCGTCCGGTCCACGTGTCGGCGTCCGGTTCGTTCCCGCGGC
v18_g21_PPD-D1                CCGTCC~~TCCACGTGTCGGCGTCCGGTTCGTTCCCGCGGC
v189_g21_PPD-D1               CCGT~~~~~CCACGTGTCGGCGTCCGGTTCGTTCCCGCGGC
v14_g21_PPD-D1                CCGTCC~~~~~~~~~~TCGGCGTCCGGTTCGTTCCCGCGGC
v33_g21_PPD-D1_1              CCGTCCGGTCCACGTGTCGGCGTCCGGTTCGTTCCCGCGGC
v33_g21_PPD-D1_2              CCGTCCAGTCCACGTGTCGGCGTCCGGTTCGTTCCCGCGGC
v75_g21_PPD-D1                CCGTCCGGTCCACGTGTCGGCGTCCGGTTCGTTCCCGCGGC
v80_g21_PPD-D1_1              CCGTCCAGTCCACGTGTCGGCGTCCGGTTCGTTCCCGCGGC
v83_g21_PPD-D1                CCGTCCTGTCCACGTGTCGGCGTCCGGTTCGTTCCCGCGGC
v122_g21_PPD-D1               CCGTCCTGTCCACGTGTCGGCGTCCGGTTCGTTCCCGCGGC
v126_g21_PPD-D1               CCGTCCAGTCCACGTGTCGGCGTCCGGTTCGTTCCCGCGGC
v127_g21_PPD-D1               CCGTCCAGTCCACGTGTCGGCGTCCGGTTCGTTCCCGCGGC
v146_g21_PPD-D1               CCGTCCAGTCCACGTGTCGGCGTCCGGTTCGTTCCCGCGGC
v9_g21_PPD-D1                 CCGTCCTGTCCACGTGTCGGCGTCCGGTTCGTTCCCGCGGC
```

Nucleotide positions are given relative to the start codon (ATG) of the *Ppd-D1* gene, with starting position corresponding to -957 bp upstream of the start codon.

Velut Mutant Lines *Ppd-D1* Short Indel Sequences around gRNA18

```
                                                  <=================  gRNA18

                                   -734      -724       -714       -704

                              ....|....|....|....|....|....|....|....|.
                                            CHE
ENA|DQ885766.1                GGATCTCTTTCTGGCCCCACC~ACCGGCGCTCCCCGCCTCC
v32_g18_PPD-D1                GGATCTCTTTCTGGCCCCAC~~~~~~~CGCTCCCCGCCTCC
v3_g18_PPD-D1                 GGATCTCTTTCTGGCCCCAC~~ACCGGCGCTCCCCGCCTCC
v11_g18_PPD-D1                GGATCTCTTTCTGGCCCCAC~~ACCGGCGCTCCCCGCCTCC
v14_18_PPD-D1_1               GGATCTCTTTCTGGCCCCAC~~ACCGGCGCTCCCCGCCTCC
v18_g18_PPD-D1_1              GGATCTCTTTCTGGCCC~~~~~ACCGGCGCTCCCCGCCTCC
v18_g18_PPD-D1_2              GGATCTCTTTCTGGCCCC~~~~ACCGGCGCTCCCCGCCTCC
v33_g18_PPD-D1_1              GGATCTCTTTCTGGCCCCAC~~ACCGGCGCTCCCCGCCTCC
v66_g18_PPD-D1                GGATCTCTTTCTGGCC~~~~~~ACCGGCGCTCCCCGCCTCC
v83_g18_PPD-D1                GGATCTCTTTCTGGCCC~~~~~ACCGGCGCTCCCCGCCTCC
v99_g18_PPD-D1                GGATCTCTTTCTGGCCCCAC~~ACCGGCGCTCCCCGCCTCC
v124_g18_PPD-D1               GGATCTCTTTCTGGCCCCACC~~CCGGCGCTCCCCGCCTCC
v126_g18_PPD-D1_2             GGATCTCTTTCTGGCCCCAC~~A~~GGCGCTCCCCGCCTCC
v146_g18_PPD-D1_1             GGATCTCTTTCTGGCCC~~~~~~~~GGCGCTCCCCGCCTCC
v146_g18_PPD-D1_2             GGATCTCTTTCTGGCCCCACACACCGGCGCTCCCCGCCTCC
v165_g18_PPD-D1               GGATCTCTTTCTGGCCCCACACACCGGCGCTCCCCGCCTCC
v164_g18_PPD-D1               GGATCTCTTTCTGGCCCCACACACCGGCGCTCCCCGCCTCC
v112_g18_PPD-D1               GGATCTCTTTCTGGCCCCACTCACCGGCGCTCCCCGCCTCC
v55_g18_PPD-D1                GGATCTCTTTCTGGCCCCACTCACCGGCGCTCCCCGCCTCC
v122_g18_PPD-D1               GGATCTCTTTCTGGCCCCACACACCGGCGCTCCCCGCCTCC
v9_g18_PPD-D1                 GGATCTCTTTCTGGCCCCACACACCGGCGCTCCCCGCCTCC
v39_g18_PPD-D1                GGATCTCTTTCTGGCCCCACACACCGGCGCTCCCCGCCTCC
v189_g18_PPD-D1               GGATCTCTTTCTGGCCCCACACACCGGCGCTCCCCGCCTCC
v21_g18_PPD-D1                GGATCTCTTTCTGGCCCCACACACCGGCGCTCCCCGCCTCC
v14_18_PPD-D1_2               GGATCTCTTTCTGGCCCCACACACCGGCGCTCCCCGCCTCC
v72_g18_PPD-D1                GGATCTCTTTCTGGCCCCACACACCGGCGCTCCCCGCCTCC
v75_g18_PPD-D1                GGATCTCTTTCTGGCCCCACACACCGGCGCTCCCCGCCTCC
v163_g18_PPD-D1               GGATCTCTTTCTGGCCCCACACACCGGCGCTCCCCGCCTCC
v80_g18_PPD-D1                GGATCTCTTTCTGGCCCCACACACCGGCGCTCCCCGCCTCC
v127_g18_PPD-D1               GGATCTCTTTCTGGCCCCACCCACCGGCGCTCCCCGCCTCC
v37_g18_PPD-D1                GGATCTCTTTCTGGCCCCACGCACCGGCGCTCCCCGCCTCC
v52_g18_PPD-D1                GGATCTCTTTCTGGCCCCACTCACCGGCGCTCCCCGCCTCC
v33_g18_PPD-D1_2              GGATCTCTTTCTGGCCCCACTCACCGGCGCTCCCCGCCTCC
v126_g18_PPD-D1_1             GGATCTCTTTCTGGCCCCACTCACCGGCGCTCCCCGCCTCC
```

Nucleotide positions are given relative to the start codon (ATG) of the *Ppd-D1* gene, with starting position corresponding to −744 bp upstream of the start codon.

Velut Mutant Lines *Ppd-D1* Long Deletion Sequences

```
                                                  <== gRNA21

                  -956      -946      -936      -926      -916      -906      -896      -886      -876      -866      -856      -846
                  ....|....|....|....|....|....|....|....|....|....|....|....|....|....|....|....|....|....|....|....|....|....|....|....|
                                     BOXIIPCCHS                                                                    CHE
ENA|DQ885766.1    AGGCGAGGGCACCCGTCCGTCCACGTGTCGGCGTCCGGTTCGTTCCCGCGGCAAGCAGCTCCTCCGCTGCCTCCGCCAGCGGCCGCCAGGCGGGGCCCACAAAATCCACATCCCCGGGCG
v8_PPD-D1         AGGCGAGGGCACCCGTCCG-----------------------------------------------------------------------------------------------------
v12_PPD-D1_1      AGGCGAGGGC------------------------------------------AAGCAGCTCCTCCGCTGCCTCCGCCAGCGGCCGCCAGGCGGGGCCCACAAAATCCACATCCCCGGGCG
v12_PPD-D1_2      AGGCGAGGGCACCCGTCC------------------------------------------------------------------------------------------------------
v46_PPD-D1_1      AGGCGAGGGCACCCGTCCGTCCACGTGTCGGCGTCCGGTTCGTTCCCGCGGCAAGCAGCTCCTCCGCTGCCTCCGCCAGCGGTCGCCAGGCGGGGCCCACAAAATCCACATCCCCGGGCG
v46_PPD-D1_2      AGGCGAGGGCACCCGTCCG-----------------------------------------------------------------------------------------------------
v59_PPD-D1_1      AGGCGAGGGCACCCGT--------------------------------------------------------------------------------------------------------
v121_PPD-D1       AGGCGAGGGCACCCGTCCG-----------------------------------------------------------------------------------------------------
v20_PPD-D1        AGGCGAGGGCACCCGTCCG-----------------------------------------------------------------------------------------------------
v71_PPD-D1        AGGCGAGGGCACCCGTCC------------------------------------------------------------------------------------------------------
v115_PPD-D1       AGGCGAGGGCACCCGTCC----ACGTGTCGGCGTCCGGTTCGTTCCCGCGGCAAGCAGCTCCTCCGCTGCCTCCGCCAGCGGCCGCCAGGCGGGGCCCACAAAATCCACATCCCCGGGCG

                  -836      -826      -816      -806      -796      -786      -776      -766      -756      -746      -736  -726
                  ....|....|....|....|....|....|....|....|....|....|....|....|....|....|....|....|....|....|....|....|....|....|....|
                                                                 CCA1                             VOZ2                              CHE
ENA| DQ885766.1   ATCCGCTGGCCCGGCCCGGGCTCGGCTCCTCCGGAGAGGAGAAAAGATATGCGGATCCAGCGCGGGTGCGGGGGCGTGGAGCGCGCGGCTTTTGATTTACGGGGATCTCTTTCTGGCCCC
v8_PPD-D1         ------------------------------------------------------------------------------------------------------------------------
v12_PPD-D1_1      ATCCGCTGGCCCGGCCCGGGCTCGGCTCCTCCGGAGAGGAGAAAAGATATGCGGATCCATGGCGGGTGCCCTCCCGTGAAGCGCGCGGCTTTTGATTTACGGGGATCTCTTTCTGGCCCC
v12_PPD-D1_2      ------------------------------------------------------------------------------------------------------------------------
v46_PPD-D1_1      ATCCGCTGGCCCGGCCCGGGCTCGGCTCCTCCGGAGAGGAGAAAAGATATGCGGATCCAGCGCGGGTGCGGGGGCGTGGAGCGCGCGGCTTTTGATTTACGGGGATCTCTTTcTGgCCCC
v46_PPD-D1_2      ------------------------------------------------------------------------------------------------------------------------
v59_PPD-D1_1      ------------------------------------------------------------------------------------------------------------------------
v121_PPD-D1       ------------------------------------------------------------------------------------------------------------------------
v20_PPD-D1        ------------------------------------------------------------------------------------------------------------------------
v71_PPD-D1        ------------------------------------------------------------------------------------------------------------------------
v115_PPD-D1       ATCCGCTGGCCCGGCCCGGGCTCGGCTCCTCCGGAGAGGAGAAAAGATATGCGGATCCAGCGCGGGTGCGGGGGCGTGGAGCGCGCGGCTTTTGATTTACGGGGATCTCTTTCTGGCCCC
                  <== gRNA18
                  -716      -706      -696      -686      -676
                  ....|....|....|....|....|....|....|....|....|....|...
                                            BOXIIPCCHS
ENA| DQ885766.1   ACCACCGGCGCTCCCCGCCTCCATTTCGTCCACGTCACCCGTCCCCCTCCCCC
v8_PPD-D1         --CACCGGCGCTCCCCGCCTCCATTTCGTCCACGTCACCCGTCCCCCTCCCCC
v12_PPD-D1_1      ACCACCGGCGCTCCCCGCCTCCATTTCGTCCACGTCACCCGTCCCCCTCCCCC
v12_PPD-D1_2      ------------------------------------------CCCCCTCCCCC
v46_PPD-D1_1      aC----------------CTCCATTtcgTCCACGTCACCCGTCCCCCTCCCCC
v46_PPD-D1_2      --CACCGGCGCTCCCCGCCTCCATTTCGTCCACGTCACCCGTCCCCCTCCCCC
v59_PPD-D1_1      ------------------------------------------CCCCCTCCCCC
v121_PPD-D1       AC-ACCGGCGCTCCCCGCCTCCATTTCGTCCACGTCACCCGTCCCCCTCCCCC
v20_PPD-D1        --CACCGGCGCTCCCCGCCTCCATTTCGTCCACGTCACCCGTCCCCCTCCCCC
v71_PPD-D1        A-CACCGGCGCTCCCCGCCTCCATTTCGTCCACGTCACCCGTCCCCCTCCCCC
v115_PPD-D1       ACC----------------TCCATTTCGTCCACGTCACCCGTCCCCCTCCCCC
```

Nucleotide positions are given relative to the start codon (ATG) of the *Ppd-D1* gene, with starting position corresponding to -966 bp upstream of the start codon.

Velut Mutant Lines *Ppd-D1* Long Insertion Sequences

```
                      -745      -735      -725      -715      -705      -695      -685      -675      -665      -655      -645      -635
                  ....|....|....|....|....|....|....|....|....|....|....|....|....|....|....|....|....|....|....|....|....|....|....|....|
                                           CHE
ENA|DQ885766.1    TTGATTTACGGGGATCTCTTTCTGGCCCCAC~~~~~~~~~~~~~~~~~~~~~~~~~~~~~~~~~~~~~~~~~~~~~~~~~~~~~~~~~~~~~~~~~~~~~~~~~~~~~~~~~~~~~~~~~ 31
v103_PPD-D1       TTGATTTACGGGGATCTCTTTCTGGCCCCACGCCGTGCCGTCTGCTTCGCTTGGCCCGACGCGACGCTGCTGGTTCGCTGGTTGGTGTCCGTTAGACTCGTCGACGGCGTTTAACAGGCT 120

                      -625      -615      -605      -595      -585      -575      -565      -555      -545      -535      -525      -515
                  ....|....|....|....|....|....|....|....|....|....|....|....|....|....|....|....|....|....|....|....|....|....|....|
ENA|DQ885766.1    ~~~~~~~~~~~~~~~~~~~~~~~~~~~~~~~~~~~~~~~~~~~~~~~~~~~~~~~~~~~~~~~~~~~~~~~~~~~~~~~~~~~~~~~~~~~~~~~~~~~~~~~~~~~~~~~~~~~~ 31
v103_PPD-D1       GGCATTATCTACTCGAAACAAGAAAAATGTTTCCTTAGTTTTTTTTGCAAATACCTTCACCTATATAATACTTCATCCATTTTATTAGTACATCCATTTAGGGTTTAGGGTTAATGGTTT 240

                       -505      -495      -485      -475      -465      -455      -445      -435      -425      -415      -405      -395
                  ....|....|....|....|....|....|....|....|....|....|....|....|....|....|....|....|....|....|....|....|....|....|....|
ENA|DQ885766.1    ~~~~~~~~~~~~~~~~~~~~~~~~~~~~~~~~~~~~~~~~~~~~~~~~~~~~~~~~~~~~~~~~~~~~~~~~~~~~~~~~~~~~~~~~~~~~~~~~~~~~~~~~~~~~~~~~~~~~ 31
v103_PPD-D1       TTATAGACTAATTTTTTTAGTACATCTATTTTATTCTATTTTAGCCTCTAAATTAAGAAAACTAAAACTCTATTTTAGTTTTTTTATTTAATAATTTAGATATAAAATAGAATAAAATAA 360
                              <==============  gRNA18
                      -385      -375      -365      -355      -345      -335      -325      -315      -305
                  ....|....|....|....|....|....|....|....|....|....|....|....|....|....|....|....|....|....|...
                                                          BOXIIPCCHS
ENA|DQ885766.1    ~~~~~~~~~~~~~~~~CACCGGCGCTCCCCGCCTCCATTTCGTCCACGTCACCCGTCCCCCTCCCCCAATATCTACTCCTCCGCCCCCCTTGG 108
v103_PPD-D1       ATGGACTAAAAATTATCaCCGGCGCTCCCCGCCTCCATTTCGTCCACGTCGCCCGTCCCCCTCCCCCGATATCTACTCCTCCGCCCCCCTTGG 453
```

Nucleotide positions are given relative to the start codon (ATG) of the *Ppd-D1* gene, with starting position corresponding corresponding to −755 bp upstream of the start codon

```
                      -1010     -1000     -990      -980      -970      -960      -950      -940      -930      -920      -910      -900
                  ....|....|....|....|....|....|....|....|....|....|....|....|....|....|....|....|....|....|....|....|....|....|....|....|
ENA|DQ885766.1    AAGGTTGAAGAAGATGGATTCAGTTGACGGCTCCCGCGAGTCAAAGGAGTTGGGAGGCGAGGGCACCCGTC~~~~~~~~~~~~~~~~~~~~~~~~~~~~~~~~~~~~~~~~~~~~~~~~~
v22_PPD-D1_1      AAGGTTGAAGAAGATGGATTCAGTTGACGGCTCCCGCGAGTCAAAGGAGTTGGGAGGCGAGGGCCCCGTCTTGCAGGCAGCAGCGGCCGAAGAAGAACACACACGAATCTTGGCCGGTAC

                      -890      -880      -870      -860      -850      -840      -830      -820      -810      -800      -790      -780
                  ....|....|....|....|....|....|....|....|....|....|....|....|....|....|....|....|....|....|....|....|....|....|....|
ENA|DQ885766.1    ~~~~~~~~~~~~~~~~~~~~~~~~~~~~~~~~~~~~~~~~~~~~~~~~~~~~~~~~~~~~~~~~~~~~~~~~~~~~~~~~~~~~~~~~~~~~~~~~~~~~~~~~~~~~~~~~~~~~
v22_PPD-D1_1      AGAGCACTTGAGAATGTATTGAGAGAAGGGAGGAACCAGATCCTGTACAGGACCACACAGGTGAATCAGAGCCGGTGGACTCCACGATATCAAGGAGTTGAGTCCACATGTCCAAAGT
                      -770      -760      -750      -740      -730      -720      -710      -700      -690      -680      -670      -660
                  ....|....|....|....|....|....|....|....|....|....|....|....|....|....|....|....|....|....|....|....|....|....|....|
ENA|DQ885766.1    ~~~~~~~~~~~~~~~~~~~~~~~~~~~~~~~~~~~~~~~~~~~~~~~~~~~~~~~~~~~~~~~~~~~~~~~~~~~~~~~~~~~~~~~~~~~~~~~~~~~~~~~~~~~~~~~~~~~~
v22_PPD-D1_1      CCCCGAAGACGCCGCCAGCCACCCGCGTGATCACCAGATTCACCATCATAGNTAATCAATTGYTCCGAACGCCTACCACGTACTCGTACTATATATATGGCGACTGAAGAGCCGGTAAGA

                      -650      -640      -630      -620      -610      -600      -590      -580      -570      -560      -550      -540
....|....|....|....|....|....|....|....|....|....|....|....|....|....|....|....|....|....|....|....|....|....|....| ....|....| ....|
ENA|DQ885766.1    ~~~~~~~~~~~~~~~~~~~~~~~~~~~~~~~~~~~~~~~~~~~~~~~~~~~~~~~~~~~~~~~~~~~~~~~~~~~~~~~~~~~~~~~~~~~~~~~~~~~~~~~~~~~~~~~~~~~~
v22_PPD-D1_1      AGAAGCTACTACAGTACGTACAAGCTAGGCGGTACGTACGTAGGATGCAGGAGCTAGCGGGCTAGCGGCATATATCCATGAGCGTTTGAGCAATGAAAATGCGGTTGCCCGTCGGCGTCC

                                                                                                <==============  gRNA21
                      -530      -520      -510      -500      -490      -480      -470      -460      -450      -440
                  ....|....|....|....|....|....|....|....|....|....|....|....|....|....|....|....|....|....|....|....|....|.
                                                                                                 BOXIIPCCHS
ENA|DQ885766.1    ~~~~~~~~~~~~~~~~~~~~~~~~~~~~~~~~~~~~~~~~~~~~~~~~~~~~~~~~~~~~~~~~~~~~~~~~~~~~~~~~~~~~~~~~~~CGTCCACGTGTCGGCG
v22_PPD-D1_1      ATGCATTCAAGCATAAGGACGTACGTACGTAGGATGCAGGAGCTAGCGGGCTAGCGGCATATATCCATGAGCGTTTGAGCAATGAAAATGCGGTTGCCCGTCGGCG
```

Nucleotide positions are given relative to the start codon (ATG) of the *Ppd-D1* gene, with starting position corresponding corresponding to −1020 bp upstream of the start codon

```
                                                                                                             <==============  gRNA21
                      -1049     -1039     -1029     -1019     -1009     -999      -989      -979      -969      -959      -949      -939
                  ....|....|....|....|....|....|....|....|....|....|....|....|....|....|....|....|....|....|....|....|....|....|....|....|
                                                                                                                        BOXIIPCCHS
ENA|DQ885766.1    GCTTCTTCACACTACTCACTGTAGTAGTAGACTACGAGTAAGGTTGAAGAAGATGGATTCAGTTGACGGCTCCCGCGAGTCAAAGGAGTTGGGAGGCGAGGGCACCCGTCCGTCCACGTG
v22_PPD-D1_2      GCTTCTTCACACTACTCACTGTAGTAGTAGACTACGAGTAAGGTTGAAGAAGATGGATTCAGTTGACGGCTCCCGCGAGTCAAAGGAGTTGGGAGGCGAGGGC~~~~~~~~~~~~~~~~~

                      -929      -919      -909      -899      -889      -879      -869      -859      -849      -839      -829      -819
                  ....|....|....|....|....|....|....|....|....|....|....|....|....|....|....|....|....|....|....|....|....|....|....|
ENA|DQ885766.1    TCGGCGT~~~~~~~~~~~~~~~~~~~~~~~~~~~~~~~~~~~~~~~~~~~~~~~~~~~~~~~~~~~~~~~~~~~~~~~~~~~~~~~~~~~~~~~~~~~~~~~~~~~~~~~~~~~~~
v22_PPD-D1_2      ~~~~~GTGCGGTTCGTTCCCGCGGCAAGCAGCTCCTCCGCTGCCTCCGCCAGCGGCCGCCAGGCGGGGCCCACAAAATTTGTTTTTTTGTTTTCTTTGTTGCTTTATTTTTTTTATTTTT

                      -809      -799      -789      -779      -769      -759      -749      -739      -729      -719      -709      -699
                  ....|....|....|....|....|....|....|....|....|....|....|....|....|....|....|....|....|....|....|....|....|....|....|
ENA|DQ885766.1    ~~~~~~~~~~~~~~~~~~~~~~~~~~~~~~~~~~~~~~~~~~~~~~~~~~~~~~~~~~~~~~~~~~~~~~~~~~~~~~~~~~~~~~~~~~~~~~~~~~~~~~~~~~~~~~~~~~~~
v22_PPD-D1_2      TTCGACTTACATCCTTTTGTAAAAGATTTTTAAGAGGCCTAGAAATTTAGAGAAGTCTTTAATAAACCTCCTATAGAAACCAGCATGACCAAGGAAACTACSAATACCTTTGATAATCTT

                      -689      -679      -669      -659      -649      -639      -629      -619      -609      -599      -589      -579
                  ....|....|....|....|....|....|....|....|....|....|....|....|....|....|....|....|....|....|....|....|....|....|....|
ENA|DQ885766.1    ~~~~~~~~~~~~~~~~~~~~~~~~~~~~~~~~~~~~~~~~~~~~~~~~~~~~~~~~~~~~~~~~~~~~~~~~~~~~~~~~~~~~~~~~~~~~~~~~~~~~~~~~~~~~~~~~~~~~
v22_PPD-D1_2      TAGGTCGGCGTCTGCTTCTTGGTGCCGCGCCTCCGCTTATAAGCTGGTTCTGCTCCCTGTAAACTGGCGAGGTGGGACAAATGTTARACGTGCTGCTGCACAGCAKCGCCAGCCCAACCA

                      -569      -559      -549      -539      -529
                  ....|....|....|....|....|....|....|....|....|....|..
ENA|DQ885766.1    ~~~~~~~~~~~~~~~~~~~~~~~~~~~~~~~~~~~~~~~~~~~~~~~~~~~~
v22_PPD-D1_2      CACCACATCATCACAACCAAGCGAACAAGAAAGCATTTCTGTATATGCTTCT
```

Nucleotide positions are given relative to the start codon (ATG) of the *Ppd-D1* gene, with starting position corresponding corresponding to −1059 bp upstream of the start codon

Velut Mutant Lines *Ppd-B1* Indel Sequences around gRNA18

```
                                                                  <------------------ gRNA18
                                 -730      -720      -710      -700    -690       -680
                      ....|....|....|....|....|....|....|....|....|....|....|....|....|..
                                                              CHE
ncbi DQ885757.1       TTTTGATTTACGGGGATCTCTTTCTGGCCGCCCCGGGGCCCCAC~~CACCGGCGCTCCCCGCCTCC
v46_g18_PPD-B1_1      TTTTGATTTACGGGGATCTCTTTCTGGCCGCCCCGgggccccac~~~~~~~~~~ctccccgcctcc
v46_g18_PPD-B1_2      TTTTGATTTACGGGGATCTCTTTCTGGCCGCCCCG~~~~~~~~~~~~~~~~~~~~~~~~~~cctcc
v75_g18_PPD-B1_1      TTTTGATTTACGGGGATCTCTTTCTG~~~~~~~~~~~~~~~~~~~~CACCGGCGCTCCCCGCCTCC
v75_g18_PPD-B1_2      TTTTGATTTACGGGGATCTCTTTCTG~~~~~~~~~~~~~~~~~~~~~~~~~~~GCTCCCCGCCTCC
v103_g18_PPD-B1_1     TTTTGATTTACGGGGATCTCTT~~~~~~~~~~~~~~~~~~~~~~~~~~~~~~~~~~~~~~~~~TCC
v103_g18_PPD-B1_2     TTTTGATTTACGGGGATCTCTTTCTGGCCGCCCCGGGGCCCCACCA~~~~~GCGCTCCCCGCCTCC
v121_g18_PPD-B1_1     TTTTGATTTACGGGGATCTCTTTCTGGCCGCCCCGGGGCCC~~~~~~~~~~~~~~~~~~~GCCTCC
v121_g18_PPD-B1_2     TTTTGATTTACGGGGATCTCTTTCTGGCCGCCCCGGGGCCCCACC~~~CCGGCGCTCCCCGCCTCC
v122_g18_PPD-B1_1     TTTTGATTTACGGGGATCTCTTTCTGGCCGCCCCGGGGCCCCAC~~~ACCGGCGCTCCCCGCCTCC
v126_g18_PPD-B1_1     TTTTGATTTACGGGGATCTCTTTCTGGCCGCCCCGGGGCCCCAC~~~ACCGGCGCTCCCCGCCTCC
v33_g18_PPD-B1        TTTTGATTTACGGGGATCTCTTTCTGGCCGCCCCGGGGCCCCAC~~~ACCGGCGCTCCCCGCCTCC
v189_g18_PPD-B1       TTTTGATTTACGGGGATCTCTTTCTGGCCGCCCCGGGGCCCCAC~~~ACCGGCGCTCCCCGCCTCC
v8_g18_PPD-B1         TTTTGATTTACGGGGATCTCTTTCTGGCCCC~~~~~~~~~~~~~ACCACCGGCGCTCCCCGCCTCC
v18_g18_PPD-B1        TTTTGATTTACGGGGATCTCTTTCTGGCCGCCCCGGGGCCCC~~~~~~~~~~~~~~~~~~~CCTCC
v146_g18_PPD-B1       TTTTGATTTACGGGGATCTCTTTCTGGCCGCCCCGGGGCCCCACC~~~~~~~~~~~~~~~~~~TCC
v121_g18_PPD-B1       TTTTGATTTACGGGGATCTCTTTCTGGCCGCCCCGGGGCCCCAC~~~ACCGGCGCTCCCCGCCTCC
v103_g18_PPD-B1_1     TTTTGATTTACGGGGATCTCTTT~~~~~~~~~~~~~~~~~~~~~~~~~~~~~~~~~~~~~~~~~CC
v103_g18_PPD-B1_2     TTTTGATTTACGGGGATCTCTTTCTGGCCGCCCCGGGGCCCCACCA~~~~~GCGCTCCCCGCCTCC
v115_g18_PPD-B1_1     TTTTGATTTACGGGGATCTCTTTCTGGCCGCCCCGGGGCCC~~~~~~ACCGGCGCTCCCCGCCTCC
v74_g18_PPD-B1_1      TTTTGATTTACGGGGATCTCTTTCTGGCCGCCCCGGGGCCC~~~~~~~~~GGCGCTCCCCGCCTCC
v32_g18_PPD-B1_2      TTTTGATTTACGGGGATCTCTTTCTGGCCGCCCCGGG~~~~~~~~~~ACCGGCGCTCCCCGCCTCC
v32_g18_PPD-B1_1      TTTTGATTTACGGGGATCTCTTTCTGGCCGCCCCGGGGCCCG~~~~~~~~~~~~~~~~CCGCCTCC
v25_g18_PPD-B1        TTTTGATTTACGGGGATCTCTTTCTGGCCGCCCCGGG~~~~~~~~~~ACCGGCGCTCCCCGCCTCC
v27_g18_PPD-B1        TTTTGATTTACGGGGATCTCTTTCTGGCCCCCCC~~~~~~~~~~~~~ACCGGCGCTCCCCGCCTCC
v52_g18_PPD-B1_1      TTTTGATTTACGGGGATCTCTTTCTGGCCGCCCCGGGGCCC~~~~~~ACCGGCGCTCCCCGCCTCC~
v10_g18_PPD-B1        TTTTGATTTACGGGGATCTCTTTCTGGCCGCCCCGGGGCCCCACTACACCGGCGCTCCCCGCCTCC
v112_g18_PPD-B1       TTTTGATTTACGGGGATCTCTTTCTGGCCGCCCCGGGGCCCCACA~CACCGGCGCTCCCCGCCTCC
v12_g18_PPD-B1        TTTTGATTTACGGGGATCTCTTTCTGGCCGCCCCGGGGCCCCACG~CACCGGCGCTCCCCGCCTCC
v124_g18_PPD-B1       TTTTGATTTACGGGGATCTCTTTCTGGCCGCCCCGGGGCCCCACA~CACCGGCGCTCCCCGCCTCC
v127_g18_PPD-B1       TTTTGATTTACGGGGATCTCTTTCTGGCCGCCCCGGGGCCCCACA~CACCGGCGCTCCCCGCCTCC
v163_g18_PPD-B1_2     TTTTGATTTACGGGGATCTCTTTCTGGCCGCCCCGGGGCCCCACA~CACCGGCGCTCCCCGCCTCC
v164_g18_PPD-B1       TTTTGATTTACGGGGATCTCTTTCTGGCCGCCCCGGGGCCCCACA~CACCGGCGCTCCCCGCCTCC
v165_g18_PPD-B1       TTTTGATTTACGGGGATCTCTTTCTGGCCGCCCCGGGGCCCCACA~CACCGGCGCTCCCCGCCTCC
v19_g18_PPD-B1        TTTTGATTTACGGGGATCTCTTTCTGGCCGCCCCGGGGCCCCACA~CACCGGCGCTCCCCGCCTCC
v21_g18_PPD-B1        TTTTGATTTACGGGGATCTCTTTCTGGCCGCCCCGGGGCCCCACT~CACCGGCGCTCCCCGCCTCC
v39_g18_PPD-B1        TTTTGATTTACGGGGATCTCTTTCTGGCCGCCCCGGGGCCCCACT~CACCGGCGCTCCCCGCCTCC
v55_g18_PPD-B1_1      TTTTGATTTACGGGGATCTCTTTCTGGCCGCCCCGGGGCCCCACA~~ACCGGCGCTCCCCGCCTCC
v55_g18_PPD-B1_2      TTTTGATTTACGGGGATCTCTTTCTGGCCGCCCCGGGGCCCCACA~CACCGGCGCTCCCCGCCTCC
v66_g18_PPD-B1        TTTTGATTTACGGGGATCTCTTTCTGGCCGCCCCGGGGCCCCACA~CACCGGCGCTCCCCGCCTCC
v71_g18_PPD-B1        TTTTGATTTACGGGGATCTCTTTCTGGCCGCCCCGGGGCCCCACA~CACCGGCGCTCCCCGCCTCC
v72_g18_PPD-B1        TTTTGATTTACGGGGATCTCTTTCTGGCCGCCCCGGGGCCCCACT~CACCGGCGCTCCCCGCCTCC
v73_g18_PPD-B1        TTTTGATTTACGGGGATCTCTTTCTGGCCGCCCCGGGGCCCCACG~CACCGGCGCTCCCCGCCTCC
v83_g18_PPD-B1        TTTTGATTTACGGGGATCTCTTTCTGGCCGCCCCGGGGCCCCACT~CACCGGCGCTCCCCGCCTCC
v9_g18_PPD-B1         TTTTGATTTACGGGGATCTCTTTCTGGCCGCCCCGGGGCCCCACT~CACCGGCGCTCCCCGCCTCC
v99_g18_PPD-B1        TTTTGATTTACGGGGATCTCTTTCTGGCCGCCCCGGGGCCCCACA~CACCGGCGCTCCCCGCCTCC
v122_g18_PPD-B1_2     TTTTGATTTACGGGGATCTCTTTCTGGCCGCCCCGGGGCCCCACA~CACCGGCGCTCCCCGCCTCC
v126_g18_PPD-B1_2     TTTTGATTTACGGGGATCTCTTTCTGGCCGCCCCGGGGCCCCACT~CACCGGCGCTCCCCGCCTCC
v115_g18_PPD-B1_2     TTTTGATTTACGGGGATCTCTTTCTGGCCGCCCCGGGGCCCCACA~CACCGGCGCTCCCCGCCTCC
v74_g18_PPD-B1_2      TTTTGATTTACGGGGATCTCTTTCTGGCCGCCCCGGGGCCCCACG~CACCGGCGCTCCCCGCCTCC
v52_g18_PPD-B1_2      TTTTGATTTACGGGGATCTCTTTCTGGCCGCCCCGGGGCCCCACT~CACCGGCGCTCCCCGCCTCC
```

Nucleotide positions are given relative to the start codon (ATG) of the *Ppd-B1* gene, with starting position corresponding corresponding to −740 bp upstream of the start codon
