# Supplementary material for: Fine tuning wheat heading time through genome editing of transcription factor binding sites in Ppd-1 gene promoter
Source: Sci Rep. 2025 Nov 26;15:42034. doi: 10.1038/s41598-025-25295-8 (PMC12657949; doi:10.1038/s41598-025-25295-8)
Supplement: Supplementary file 2 — Supplementary Material 2 [file 41598_2025_25295_MOESM2_ESM.pdf]

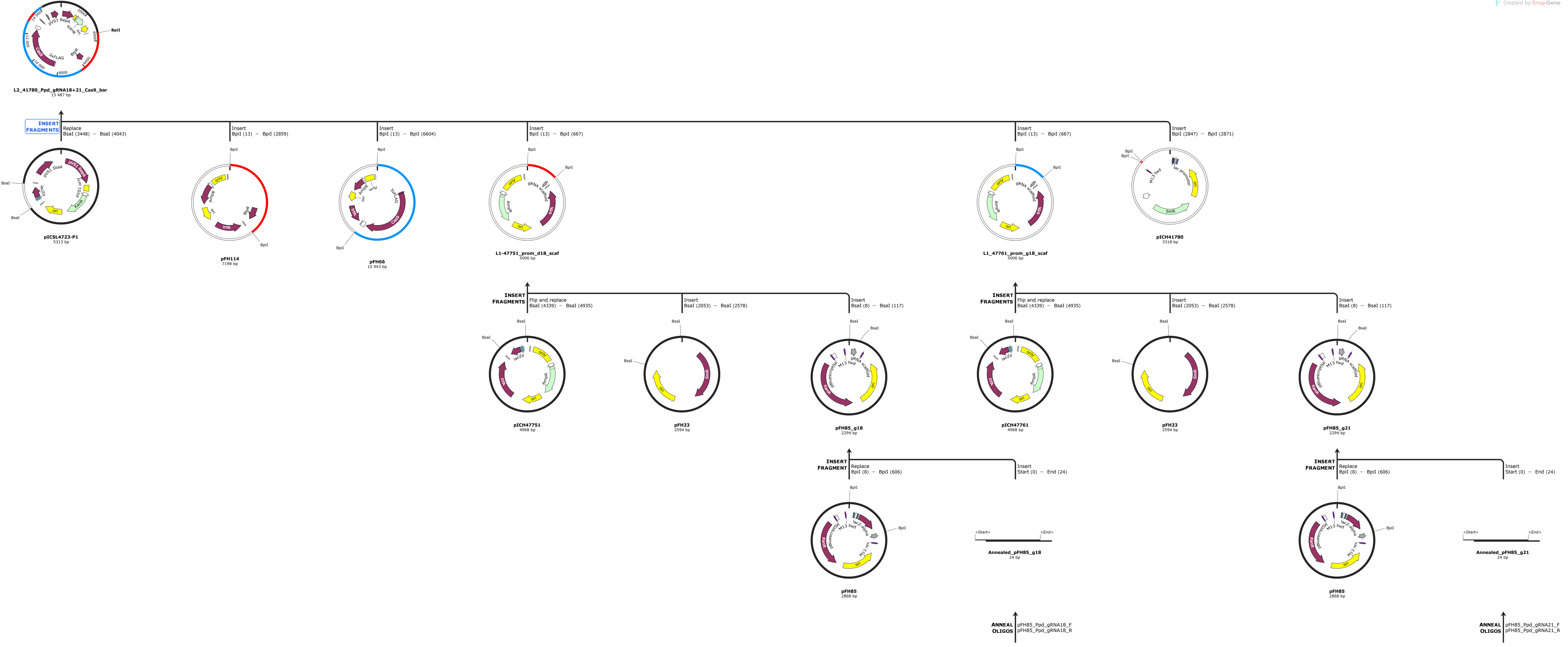


**Figure S1.** Scheme of the L2_41780_Ppd_gRNA18+21_Cas9_bar construction.

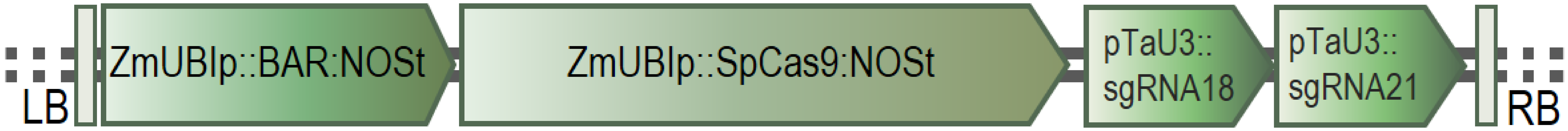


**Figure S2.** Schematic structure of plasmid L2_41780_Ppd_gRNA18+21_Cas9_bar.
